# What do we know about community-based health worker programs? A systematic review of existing reviews on community health workers

**DOI:** 10.1186/s12960-018-0304-x

**Published:** 2018-08-16

**Authors:** Kerry Scott, S. W. Beckham, Margaret Gross, George Pariyo, Krishna D Rao, Giorgio Cometto, Henry B. Perry

**Affiliations:** 1 Department of International Health, Johns Hopkins Bloomberg School of Public Health, 615 North Wolfe Street, Baltimore, 21205 United States of America; 2 Department of Health, Behavior and Society, Johns Hopkins Bloomberg School of Public Health, Baltimore, 21205 United States of America; 3 Welch Medical Library, Johns Hopkins Medical Institutions, 1900 E Monument Street, Baltimore, 21205 United States of America; 4 Health Workforce Department, World Health Organization, Avenue Appia 20, 1202 Geneva, Switzerland

## Abstract

**Objective:**

To synthesize current understanding of how community-based health worker (CHW) programs can best be designed and operated in health systems.

**Methods:**

We searched 11 databases for review articles published between 1 January 2005 and 15 June 2017. Review articles on CHWs, defined as non-professional paid or volunteer health workers based in communities, with less than 2 years of training, were included. We assessed the methodological quality of the reviews according to AMSTAR criteria, and we report our findings based on PRISMA standards.

**Findings:**

We identified 122 reviews (75 systematic reviews, of which 34 are meta-analyses, and 47 non-systematic reviews). Eighty-three of the included reviews were from low- and middle-income countries, 29 were from high-income countries, and 10 were global. CHW programs included in these reviews are diverse in interventions provided, selection and training of CHWs, supervision, remuneration, and integration into the health system. Features that enable positive CHW program outcomes include community embeddedness (whereby community members have a sense of ownership of the program and positive relationships with the CHW), supportive supervision, continuous education, and adequate logistical support and supplies. Effective integration of CHW programs into health systems can bolster program sustainability and credibility, clarify CHW roles, and foster collaboration between CHWs and higher-level health system actors. We found gaps in the review evidence, including on the rights and needs of CHWs, on effective approaches to training and supervision, on CHWs as community change agents, and on the influence of health system decentralization, social accountability, and governance.

**Conclusion:**

Evidence concerning CHW program effectiveness can help policymakers identify a range of options to consider. However, this evidence needs to be contextualized and adapted in different contexts to inform policy and practice. Advancing the evidence base with context-specific elements will be vital to helping these programs achieve their full potential.

**Electronic supplementary material:**

The online version of this article (10.1186/s12960-018-0304-x) contains supplementary material, which is available to authorized users.

## Background

Community-based health worker (CHW) programs are undergoing a resurgence, as these health workers are envisioned to be culturally adept members of comprehensive and people-centered primary health care teams that will enable universal health care [1]. The last decade has seen both the introduction as well as the re-invigoration of national CHW programs in many low- and middle-income countries (LMICs) [2, 3]. These programs involve the delivery of community-based health services by paid or volunteer health workers with fewer than 2 years training. There is a rapid growth of evidence on the effectiveness of community-based interventions [4, 5], positive experiences with reinvigorated national CHW programs [2], and renewed interest in stronger national CHW programs [6]. Health systems in LMICs and high-income countries (HICs) are expanding their utilization of CHWs in order to meet population health needs, improve access to services, address health inequities, and improve health system performance and efficiency [7]. Policymakers need evidence-based guidance to further develop this cadre of the health workforce. As a first step in developing policy guidance on health policy and systems support to optimize CHW programs, the World Health Organization (WHO) commissioned a systematic review of available reviews related to CHWs.

This systematic review synthesizes existing reviews on CHWs in order to map what is known about these programs. We present evidence on the roles and capacities of CHWs as well as the health system enablers that can support their functionality. We reviewed heterogeneous evidence to identify the types of interventions that CHWs provide, as well as optimal approaches to training, support, supervision, and remuneration, and health system integration (i.e., recognition in national health care planning, regulation, and implementation) [8].

## Methods

### Search strategy

We searched for articles published between 1 January 2005 and 15 June 2017 in 11 electronic databases: PubMed, Embase, PASCAL Biomed, the Cochrane Library, Ovid’s Global Health, WHO Global Health Regional Libraries, the Database of Abstracts of Reviews of Effects (DARE), Epistemonikos, Health Systems Evidence, PROSPERO, and the National Guideline Clearinghouse of the US Department of Health and Human Services. Searches were developed and conducted by an academic librarian (co-author MG) and peer reviewed by a second librarian prior to implementation.

The systematic literature search used a combination of controlled vocabulary and keywords for two concepts: (1) reviews and (2) community-based health workers (e.g., “community health worker”, “lay health worker”, “close-to-community provider”). We used the validated systematic review filter for PubMed [9] and expanded it to catch 30 key articles. Similarly for Embase, we used the validated Wilcynski and Haynes, “small drop in specificity, substantive gain in sensitivity” systematic review query [10] and expanded it with additional terms (metanalysis; review:ti), to include, for example, all titles with the word “review” in them. In the other nine databases, we did not use pre-developed review filters but instead used simpler search strings for the concept “review.” We did not limit to language. All titles and abstracts relevant to our study were retrieved and searched for full text. See Additional file 1 for the full PubMed search strategy.

### Eligibility criteria, screening, and article selection

Articles were included if they were (a) reviews and (b) focused on CHWs. We included systematic reviews as well as non-systematic reviews (such as realist, narrative, scoping, and literature reviews), because many non-systematic reviews provided insight into CHW program design and health system integration. Our inclusive approach brought together reviews on CHWs that used a wide range of synthesis methods to comment on many features of CHW programs, going beyond the effectiveness focus of systematic meta-analysis. We defined CHWs as health workers based in communities (i.e., conducting outreach from their homes and beyond primary health care facilities or based at peripheral health posts that are not staffed by doctors or nurses), who are either paid or volunteer, who are not professionals, and who have fewer than 2 years training but at least some training, if only for a few hours. Adhering closely to this definition led us to include some programs, such as those for peer supporters and traditional birth attendants with some training, that reflect divergent and context-specific understandings of the term “CHW.” We excluded articles that did not directly mention CHWs or mentioned them only in passing without information on their role. Article titles and abstracts were divided and assigned for independent review to two authors from among KS, HBP, SWB, KDR, and GP, with a third author from among the same group selected on a revolving basis to resolve disputes. Full texts of retained articles underwent a final screening for eligibility.

### Data extraction and quality assessment

Included articles were divided among KS, HBP, SWB, KDR, and GP for detailed data extraction. Data extractors used a pilot-tested framework (in Excel) that synthesized content on the following topics, adapted from the 2006 World Health Report’s framework on the working life of health care providers [11]: CHW roles and capacities, training, deployment, performance measurement, remuneration and incentives, support and supervision, cost effectiveness, community embeddedness, logistical support and supplies, and integration into health systems. KS spot-checked the data extraction by frequently returning to original articles for verification.

Two authors (SWB and MG) assessed the methodological quality of the systematic reviews using the 11-item validated Assessing the Methodological Quality of Systematic Reviews (AMSTAR) criteria [12]. They began by both rating the same 10 systematic reviews and then compared and discussed their ratings to obtain consensus on how to proceed. They then divided the remaining systematic reviews between them and rated a random sample of 10% in duplicate to check agreement. Disagreements were limited and resolved through discussion. For two AMSTAR items, we assessed the articles according to the original (strict) AMSTAR criteria and also for adapted (relaxed) criteria that we developed to more appropriately assess the quality of included systematic reviews. See Additional file 2 for an explanation of the ratings. The non-systematic reviews used a diverse range of non-systematic approaches to evidence synthesis across a wide array of research questions, making the application of a standardized quality criteria inappropriate.

Throughout this report, we use the term CHW although many review articles and individual studies used different terms such as close-to-community provider or trained traditional birth attendant.

## Results

**Fig. 1:**
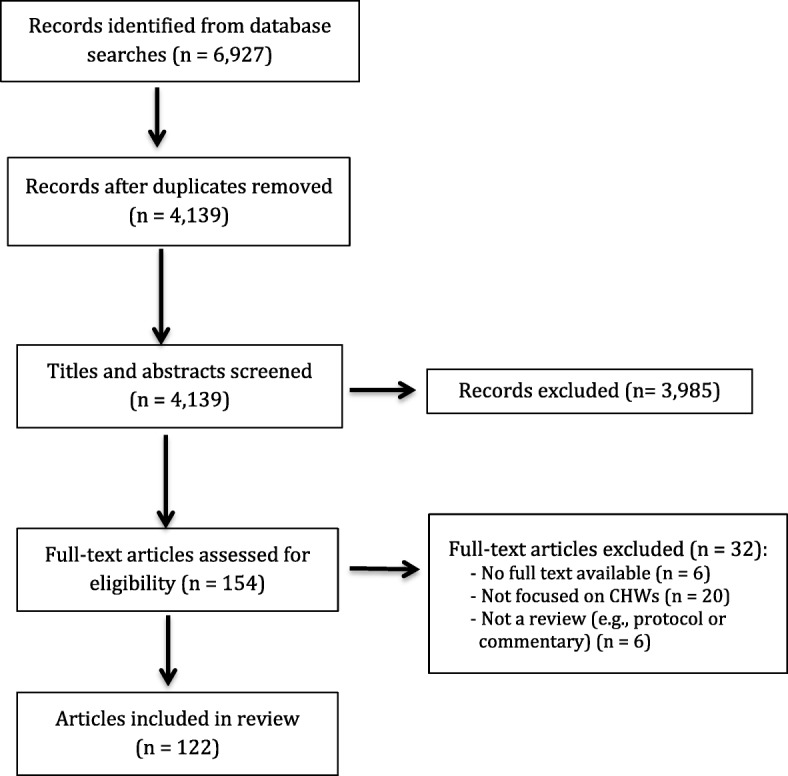
Diagram of review selection process

From 4 139 unique references identified in our search, 122 reviews met our inclusion criteria (Fig. 1). Additional file 3 provides an overview of the included reviews, which can be searched and filtered for regional focus, review type (non-systematic, systematic, meta-analysis), population focus, health issue, nature of the intervention, findings on CHW capacities and/or intervention outcomes, and AMSTAR rating. Additional file 4 presents a summary of the main findings of all included articles. Additional file 5 presents complete references of included and excluded articles.

Of the 122 included reviews, 75 were systematic (including 34 meta-analyses) and were assessed using the AMSTAR quality criteria (Additional files 2 and 3). Seven of the 11 AMSTAR indicators of quality were met by the vast majority of the systematic reviews included in our study, while the remaining four AMSTAR quality indicators (duplicate data screening and data extraction, gray literature searched, publication bias discussed; and included and excluded studies listed) were less commonly met.

Most of the reviews focused on LMICs (*n* = 83) and a range of primary health care (*n* = 14), child health (*n* = 13), and maternal and child health (*n* = 14) interventions. High-income country reviews (*n* = 29) tended to focus on non-communicable diseases (*n* = 12) and reaching specific underserved groups (*n* = 7) (Table 1).

**Table 1:** Health topics discussed in the included reviews

Focal health issue	Regional focus of studies included in the review			
	LMICs	HICs	LMICs and HICs	Total
System-level/multiple/general				
• Multiple primary health care interventions	14	1	0	15
• Health system*	7	3	0	10
• Underserved groups (e.g., Latinos in the USA)	0	7	0	7
• CHW rights/well-being	3	0	0	3
Maternal and child health				
• Child/neonatal health	13	1	0	14
• Maternal and child/neonatal health	14	0	1	15
• Vaccination	4	0	1	5
• Maternal health**	3	1	0	4
• Contraception	3	0	0	3
• Breastfeeding	0	1	1	2
Disease-specific: non-communicable				
• Diabetes	0	5	0	5
• Cancer	1	3	0	4
• Mental health**^, #^	4	2	0	6
• Other (pediatric chronic disease^#^, vascular disease, hypertension)	1	2	2	5
Disease-specific: infectious				
• HIV^#^	6	0	4	10
• Malaria	6	0	0	6
• Other infection (Buruli ulcer, tuberculosis, hepatitis B and C, neglected tropical disease)	3	1	0	4
Other (adolescent health, palliative care, physical activity promotion)	1	2	1^##^	4
Total	83	29	10	122

*LMIC* low- and middle-income country, *HIC* high-income country

*CHW program scale up [23]; CHW program integration [38]; peer telephone calls for multiple health issues [69]; intervention design factors that influence CHW performance [15]; role of allied health assistants in the health system [70, 71]; the dimensions of lay health worker programs [13]

**Two articles on maternal mental health are classified under maternal health [72, 73]

^#^One review on pediatric chronic disease had no regional focus and included only non-communicable chronic diseases (asthma, diabetes, obesity and failure to thrive) [56]; another review was specific to childhood asthma [57]; one study on adult chronic disease in South Africa primarily dealt with HIV, so is classified under “HIV”, but we note that five of the 29 articles in that review were on mental health [74]

^##^For one review (on CHWs for palliative care) [75], no articles met the inclusion criteria but the search included LMICs and HICs

We now present findings from the reviews on considerations for CHW programmatic design and operation in health systems. We first present evidence on CHW functions and their contributions to improving health outcomes. We then report on health system enablers that can support CHW functionality, including optimal approaches to training, support, supervision, remuneration, and health system integration.

### CHW roles and capacities

CHWs perform a variety of health system functions, which can be clustered into six general categories (Table 2).

**Table 2:** Health system functions of CHWs

General category of CHW function	Specific functions mentioned in reviews
1. Deliver diagnostic, treatment, and other clinical services	
	• Identify and assess sick community members: Use rapid tests for malaria [21, 61, 76] and HIV [32, 77]; determine if a child’s breathing is dangerously rapid [78], identify high-risk pregnancies [42]; monitor clinical symptoms and signs of drug toxicity in people living with HIV and refer when appropriate [52], monitor the effects of mental-health-related medications [54]; conduct breast-cancer screening exams [79], measure and monitor blood pressure [43]
	• Provide medicines and other pharmaceuticals: Dispense contraceptives [80]: administer injectable contraceptives [81]; distribute antiretroviral drugs [52], iron folic acid tablets [82], vitamin A [82] or antimalarials [33, 82]
• Directly provide care and treatment	• Directly provide care and treatment: Perform home deliveries [15, 22, 83]; vaccinate children [16]; provide community-level diagnosis and treatment for pneumonia, malaria, and other infectious diseases [84]
2. Assist with appropriate utilization of health services, make referrals	Help ethnic minorities in the USA make and keep medical appointments for cancer screening [87] or for diabetes management [88], help people with hypertension in the USA access health insurance [43], help pregnant women with birth planning and preparedness to facilitate institutional delivery [91, 92], mobilize communities around maternal and neonatal health practices [93], refer women to health facilities for delivery [82, 94], encourage access and adherence to HIV care [32, 39, 95, 96], or find underserved groups and encourage them to have their children immunized [20]
3. Provide health education and behavior change motivation to community members	Provide education to reduce HIV stigma [50] or promote behaviors that reduce risk of acquiring HIV [74]; assist with family planning [80], depression, or assessment of child mental development [97]; encourage physical activity among those with non-communicable disease [98]; promote exclusive breastfeeding [82], antenatal and postnatal care and family planning [82]; advise on tetanus vaccination [82] or family planning [82]; provide education on cancer [87, 99], hypertension [43] and diabetes [89, 100]; reduce childhood asthma-triggering behaviors and environmental pathogens that provoke asthma [56, 57]
4. Collect and record data	Perform general clerical duties [70] and data collection (including using mHealth tools [101, 102]), identify and report on malaria outbreaks [44], monitor medicine stocks and notify government agencies when stocks are low [44, 76]
5. Improve relationships between health services and communities	Act as mediators between individuals and health services (e.g., to improve provider responsiveness to patient needs) [43], act as cultural mediators [51] (e.g., between Aboriginals and non-Aboriginals in Australia [45]), serve as patient advocates (e.g., for those with diabetes [89, 90] or cancer [87] in the USA, or for mental healthcare in LMICs [51, 103]); serve as community advocates (e.g. for Latino communities in the USA [104])
6. Provide psychosocial support	Form support groups for people with HIV [14, 50] or women [93, 105]; provide anti-retroviral treatment adherence reminders [50]; provide one-to-one psychosocial support to reduce maternal depression [73, 106], for people with hypertension [43], or for USA Latino parents of youth with mental health issues [106]; support adherence to drug regimens by sending short messages to cell phones to remind people living with HIV to take their medication [107]

The number, complexity, and range of functions CHWs perform vary substantially among programs according to context-specific needs and opportunities; functions also evolve over time [13]. While there is no optimal set of tasks or workload level that maximizes CHW productivity, one review [14] cited studies that found that too many responsibilities reduce CHW productivity and service quality, and CHWs in these situations are forced to choose which tasks to perform based on factors such as feasibility, remuneration, or preference. The authors of this review conclude that CHWs are more likely to succeed when they have a clear role and a limited number of tasks. In LMICs, CHWs commonly provide curative services, and there is some evidence that being tasked with curative tasks as opposed to solely providing health education or psychosocial support may increase CHW motivation in LMIC settings [15].

Among the reviews that assessed CHW contributions to addressing specific health issues, most found that CHWs can improve health outcomes (Table 3) but many noted concerns about the low quality of included studies and emphasized the importance of health systems enablers such as training and support, discussed in later sections of this article. The reviews were heterogeneous, examining diverse CHW programs and analyzing effectiveness across a range of health outcome measures. As a result, a meta-synthesis across the reviews was unfeasible. Thus, while Table 3 summarizes evidence on CHW contributions to health outcomes, we encourage readers to refer to each individual review in Additional file 3 and Additional file 4 for details.

**Table 3:** CHW capacities for delivering specific health interventions

Health issue	Setting	
	High-income countries	Low- and middle-income countries
Multiple primary health care interventions	Most CHW programs focused on underserved populations in HICs (such as ethnic/racial minorities, economically marginalized, rural populations or immigrant groups) [25, 45, 90, 104, 106, 108, 109]. CHW interventions, such as through peer-support telephone calls [69] or home visits [110], can be effective for a wide range of health issues, including increasing knowledge about parenting [110], disease prevention (moderate strength of evidence) [25], influenza prevention [110], promotion of home safety [110], increasing parenting self efficacy [110], patient enrollment in research [99], uptake of early intervention services [99], increasing access to primary health care for screening [108], improving workplace safety (low strength of evidence) [25] and disease prevention (mixed evidence) [25], and reducing urgent care visits [110]. CHWs can reduce obesity among postpartum teens [110], improve nutritional eating habits [99]; and increase physical activity [98].	CHW programs can promote equity of healthcare access and utilization by reducing inequities relating to place of residence, gender, education and socio-economic position, and supporting more equitable uptake of referrals [111] (low-quality evidence from Brazil [112]). Deploying lay refugees/internally displaced persons as CHWs to provide basic health services to women, children, and families in camps can increase service coverage, knowledge about disease symptoms and prevention, uptake of treatment and protective behaviors, and access to reproductive health information (some evidence, weak quality) [113]. There was no clear evidence for equitable quality of services provided by CHWs, and there was limited information regarding the role of CHWs in generating community empowerment to respond to social determinants of health [111]. There is some evidence (moderate quality) that CHWs are effective in providing health education [114] and psychosocial support [114]. There is an absence of evidence on CHW potential to support community-based palliative care [75].
Reproductive, maternal, neonatal and child health		
Neonatal/child health	CHW interventions can be effective in increasing infant-stimulating home environment scores [110], reducing psychiatric diagnoses among children [110], improving child development [99], and improving child well-being (mixed evidence) [25].	CHWs providing community-based care for infants and children in resource-limited settings can reduce neonatal, infant and child mortality and morbidity (e.g., from malaria, pneumonia and diarrhea) [35, 42, 46, 84, 85, 91, 93, 115–118]. While there is high-quality evidence that home-based neonatal care reduces neonatal and perinatal mortality in South Asian settings with high neonatal mortality rates and poor access to health facility-based care [91, 116] other reviews reported mixed results, with some individual empirical studies included in reviews not showing improvements in CHW intervention areas [85]. Evidence of the impact of CHW interventions on neonatal outcomes is promising but of moderate quality [46] and on CHW capacity to provide skilled birth care is of low quality [46]. Antenatal and neonatal practice indicators significantly improved [116]. Compared to physicians, trained CHWs may screen for possible bacterial infection in young infants with relatively high sensitivity but somewhat lower specificity [119]. There is some evidence of moderate quality that CHWs are effective in the promotion of essential newborn care [114], including skin-to-skin care for newborns [114]. CHWs can perform effective case management of child pneumonia [76], although pneumonia management performance is mixed when pneumonia management is integrated with malaria diagnosis and treatment [33]. The use of CHWs, compared to usual healthcare services, may increase the number of parents who seek help for their sick child [118]. Women’s groups (facilitated by CHWs) practicing participatory learning and action, compared with usual care, have a positive impact on reducing neonatal mortality in low-resource settings (but no evidence of impact on reducing stillbirths) [105]. Trained traditional birth attendants (TBAs) compared to untrained TBAs showed significant increases in safe delivery practices and appropriate referral knowledge and practice [94] and are associated with small but significant decreases in perinatal mortality and neonatal mortality due to birth asphyxia and pneumonia [94]. However, another review [82] concludes that there is insufficient evidence to establish the potential of TBA training to improve perinatal and neonatal mortality. CHWs in Brazil have demonstrated effectiveness in increasing the frequency of child weighings [112].
Maternal health	Peer-support can be effective for reducing depressive symptoms in mothers with postnatal depression [69] and can positively impact women’s perinatal mental health [72]. One study on addressing stress and mental health among pregnant women on Medicaid in the USA found that adding a CHW to a nurse home visit program increased the number of at-risk women reached [106].	One review reported that almost all of the intervention studies involving CHWs showed a significant impact on reducing maternal mortality and on improving perinatal and postpartum service utilization indicators [35]. Another found that community-based intervention packages, which almost always involved CHWs, may have a possible effect on reducing maternal mortality, although the pooled result just crossed the line of no effect [93]. Women’s groups (facilitated by CHWs) practicing participatory learning and action, compared with usual care, have a positive impact on reducing maternal mortality in low-resource settings [105]. In settings characterized by high mortality and weak health systems, trained TBAs can contribute to reducing mortality through participation in key evidence-based interventions [94]. There is some evidence of moderate quality that CHWs are effective in providing psychosocial support [114]. CHWs were effective in delivering psychosocial and educational interventions to reduce maternal depression [73]. Non-specialist providers (a classification that includes CHWs) may be effective in reducing perinatal depression [54].
Immunization	CHW programs increase the number of children whose vaccinations were up to date (moderate quality) [16].	There is evidence, but low quality or inconsistent, that CHWs can increase immunization coverage through promoting vaccination [16, 94, 118, 120] and providing vaccination themselves [16]. There is low-quality evidence that health professionals are confident that CHWs can deliver vaccines or other medicines using compact pre-filled auto-disposal devices [121].
Contraception	CHW interventions have been found to reduce unplanned repeat births among adolescents [110, 122] but there was no significant association detected in terms of repeated pregnancies [122].	CHWs were able to deliver injectable contraception safely and effectively, with high quality and with high levels of patient satisfaction [81, 123], and initiate their use (which involves screening women and counseling them on side effects), with no difference in the quality of counseling on side effects between CHWs and clinic-based providers [81]. Most (93%) studies indicated that CHW family planning programs increased the use of modern contraception and most (83%) reported an improvement in knowledge and attitudes concerning contraceptives [80]. CHWs can provide counseling on contraceptives, provide contraceptives, and refer to health facilities for more specialized care [80].
Breastfeeding	CHW interventions can be effective for increasing breastfeeding continuation [58, 69], attempts and duration [110], initiation, duration, and exclusivity [124].	The use of lay health workers, compared to usual healthcare services, probably increases breastfeeding [118] and there is some evidence of moderate quality that CHWs are effective in exclusive breastfeeding promotion [114]. CHWs in Brazil have demonstrated effectiveness in increasing the prevalence of breastfeeding [112] and delaying the introduction of bottle feeding [112].
Non-communicable diseases (NCDs)		
Diabetes	There is weak evidence that CHW interventions improve knowledge of medication-label reading among diabetics [25]; improve self-management [60] (low strength of evidence) [25]; decrease glycaemia [60] (mixed evidence) [90] (modest reduction) [125]. There is no evidence that telephone interventions provided by lay and peer-support workers improve mental health or quality of life among diabetics [60]. For children with type 1 diabetes, CHWs improved glycemic control and decreased hospitalizations [56].	CHW capacity in addressing diabetes in LMICs was not reported in the systematic review literature.
Cancer	CHW interventions (peer support phone calls [69], home visits [110]) can be effective in increasing cancer screening rates [69, 99, 108, 110, 126]; knowledge about prostate cancer (but not screening) [110]; cancer screening (moderate evidence) [25]; planned use of cancer screening tests (mixed evidence) [25]; breast self-examination (mixed evidence) [25].	Only one non-systematic review [79] discussed the potential of CHW to address cancer in LMICs, and did not provide evidence on CHW capacity.
Mental health	CHW interventions can reduce depression [110] and stigma toward depression treatment (one study) [106], improve depression knowledge and efficacy to seek treatment [106], and produce beneficial changes in health status measures in many, but not all, studies [127]. CHW interventions in children with chronic conditions may lead to modest improvements in parental psychosocial outcomes [56] and parental quality of life [56].	CHW-led interventions can reduce the burden of mental, neurological and substance-use disorders, including depression and post-traumatic stress disorder among adults (evidence from 3 studies) [97]; and can also improve child mental health outcomes (evidence from four studies) [97]. Non-specialist providers, usually CHWs, are more effective than usual care or delayed treatment (waitlisted) groups in the provision of mental health treatments, generally for depression or post-traumatic stress [128]. Non-specialist health workers, which in this review [54] included both professionals (e.g., doctors, nurses, and social workers) and CHWs (22 of the 38 studies), compared with usual healthcare services, have some promising benefits in improving outcomes for general and perinatal depression, post-traumatic stress disorder and alcohol-use disorders, and outcome for patients with dementia and their caretakers (evidence mostly of low or very low quality) [54].
Asthma	Peer-support telephone calls can be effective for increasing the number of asthma-free days [110] as well as the use of bedding encasements for asthma patients (moderate strength of evidence) [25]. While some CHW interventions for children with asthma decreased rapid breathing episodes, activity limitation, and asthma exacerbations, and increased the number of symptom-free days, results were inconsistent and risk of bias was often unclear [56]. Lay and peer interventions for adolescents with asthma could lead to small improvements in asthma-related quality of life (weak evidence) but there was insufficient evidence on asthma control, exacerbations and medication adherence [129].	CHW capacity in addressing asthma in LMICs was not reported in the systematic review literature.
Other NCDs (chronic disease, hypertension)	Peer-support telephone calls can be effective for diet change in post-myocardial infarction patients [69]. CHW interventions may improve chronic disease management among children (modest improvements in reduced urgent care use [56], decreased symptoms [56], and fewer missed work and school days [56]) and adults [108], including improvements in blood pressure among adults with hypertension [43, 99], in self-management behaviors (including appointment keeping and adherence to antihypertensive medications [43]), and in healthcare utilization (e.g., fewer emergency visits and an increased proportion of patients having a nurse or physician) [43].	CHW capacity in addressing other NCDs in LMICs was not reported in the systematic review literature.
Infectious diseases		
HIV	Task shifting to CHWs may enhance emotional support and increase retention in care, and better link people with HIV to care (one qualitative study) [39, 95, 96].	Task shifting from higher-level providers and clinic-based care to CHWs was generally acceptable to individuals living with HIV [39, 95]. This may enhance dignity and quality of life [50] and increase retention in care [50, 95], without decreasing the quality of care [52] or patient outcomes (such as virologic failure and mortality) [50, 53, 107]. Task shifting and community-based outreach involving CHWs effectively links people living with HIV to care [96].
Malaria	CHW capacity in addressing malaria in HICs was not reported in the systematic review literature.	There is some evidence of moderate quality that CHWs are effective in malaria prevention [35, 114]. CHWs can perform rapid diagnostic tests with high sensitivity and specificity, and display high levels of adherence to treatment guidelines [21, 33, 61, 76, 86]. There was insufficient research to enable an effect on morbidity or mortality to be estimated [21].
Other infections	Home visits from CHW can be effective in increasing hepatitis B testing [110] and increasing hepatitis B virus testing uptake (moderate quality evidence) [109].	CHW interventions have helped decrease the incidence of tuberculosis [35] and contributed to the control of neglected tropical diseases [130]. They can support the control of Buruli ulcer in sub-Saharan Africa [47]. CHWs probably increase the number of people with tuberculosis who are cured, though they do not appear to affect the number of people who complete preventive therapy [118].

Lassi et al. [93] included 26 studies on community-based interventions for maternal health, of which only one was from a HIC (Greece). Chapman et al. [124] included 26 studies on breastfeeding, of which only one was from an LMIC (Mexico). Raphael et al. [56] included 17 studies on pediatric chronic disease, of which all appear to be from the USA although this is not specified. Kew et al. [129] included five studies on adolescent asthma, of which three were from HICs, while the remaining two were from Jordan. Costa et al. [98] included 26 studies on physical activity promotion, of which only one was from an LMIC (Brazil)

As shown in Table 3, CHWs can make important contributions to improving health, particularly in extending care to underserved groups, and can successfully handle complex health counseling and biomedical tasks. However, CHWs can only meet their potential in performing these roles and improving health outcomes when supported by a range of health system enablers, discussed next.

### Training

The proper amount and type of training required by CHWs must be understood in relation to the health system context, the CHWs’ pre-existing capacities, and the roles that CHWs are expected to play. Table 4 presents findings from the review literature on core considerations in CHW training domains.

**Table 4:** Summary of findings on CHW training

Topic	Summary of findings
Link between CHW training and performance (knowledge, skills, and motivation)	All nine studies in one review that described CHW training reported improvements in CHWs knowledge or skills [25]. TBA training was found to be associated with significant increases in TBA knowledge, improved attitude, behavior and advice for antenatal care, and improved pregnancy outcomes [22, 82]. Training and supervision are vital for high-quality performance in integrated community case management programs [27]. Although no studies included in Kok et al.’s review examined the impact of CHW training on health outcomes, training was found to influence CHW motivation, job satisfaction, and performance in the following ways [15]: • Training generally resulted in expanded CHW knowledge and performance • Training linked to allowances and favoritism could lead to demotivation • Continuous training increased job satisfaction/motivation • Training should include counseling and communication skills • Training can increase community confidence in CHWs
Beneficial approaches to training (e.g., continuous education and mixing of training components)	For CHW training to improve CHW performance it must include a mix of approaches (knowledge- and skills-based) [15, 21, 48], be complemented by ongoing field-based mentoring and back-up support, [15, 20, 21] and enable CHWs to have an increased sense of self-efficacy, mastery of the tasks, and self-esteem [15, 48]. In CHW programs for common peripartum mental disorders in women in LMICs, continuous supervision was found to be more effective than one-off training [73]. However, the frequency of refresher training had no effect on guideline adherence [15] and training duration had no consistent effect on the effectiveness of the intervention [24, 42]. CHW technical competency tends to drop after training, necessitating follow-up and regular supervised practice opportunities [40, 131].

Training should seek to impart both technical competency and socially oriented capacities such as skills in communication and counseling as well as awareness of the importance of confidentiality [15–17]. Awareness of the social and political determinants of health [18] and problem-solving skills were also identified as being important [19]. One review noted that theoretical, classroom-based competency-oriented CHW training to promote immunization in India is an inappropriate approach [20]. Other reviews suggest that some competencies such as record keeping or correctly interpreting malaria test results can be introduced in the classroom but require supportive supervision and hands-on practice to be implemented properly in the field [20, 21].

Training increases CHW knowledge and skills [22] and can positively influence CHW motivation, job satisfaction, and performance [23, 24]. However, there was no direct evidence linking training to health outcomes in one review that looked for it [25], nor is there evidence that different aspects of training or different training approaches affect CHW performance [24]. One pathway through which training can contribute to CHW motivation is by increasing community confidence in their CHWs and ultimately increasing CHWs’ confidence in their capacity to perform their duties [20, 24]. Relatedly, short and insufficient training erodes CHW confidence and reduces community trust and uptake of their CHW’s services [26].

### Supervision

Supervision was often mentioned as critical for the effectiveness of CHWs, and there is some evidence regarding the benefits of supervision on CHW performance [14, 15, 23, 27, 28]. However, few details of the supervisory structure (type of supervisor, frequency of supervision, and type of training and support provided to supervisors) contributing to success were mentioned [15], and few studies have tested which approaches work best or how they are best implemented [15, 29–31]. Poor-quality supervision and low recognition from the health system can undermine community embeddedness and reduce CHW motivation [32–34]. Negative interactions of CHWs with higher-level health system actors (such as punitive supervision styles) can discourage and demotivate CHWs [33]. Supervision is often one of the “weakest links” in a CHW program, and CHW programs commonly give inadequate attention to ensuring high-quality supervision [14, 35], with negative implications for CHW empowerment [36]. Table 5 summarizes findings from the review literature on support and supervision.

**Table 5:** Summary findings on supervision for CHWs

Topic	Summary of findings
Supervision appears to be effective in combination with other supports	• Supervision is critical to maintain quality and motivation [19, 23, 33, 35, 76, 132]. • In integrated community case management programs, supervision and on-site training of CHWs improved clinical practices, with providers showing increased knowledge, increased effectiveness in promoting care-seeking behaviors, or improved basic disease management [27]. • Frequent supervision and continuous training led to better CHW performance in certain settings, but the evidence is mixed [15].
Many unknowns and need more research	• There is some evidence of benefit for health care performance, but evidence quality is low [30] and follow-up is limited [29]. • Supervision and training were often mentioned as facilitating factors, but few studies have tested which approaches work best or how these were best implemented [15].
What might work?	• Supervision that focuses on supportive approaches, quality assurance and problem solving may be most effective at improving CHW performance (as opposed to more bureaucratic and punitive approaches) [15, 29, 31]. • Enhanced supervision of CHWs was only superior to routine supervision in two low quality-studies, which examined the effect of regular, supportive supervision and the use of checklists on workforce performance [30]. • Less-intensive supervision of CHWs in one study of low quality did not show any adverse effect on the quality of care or health workers attrition [30]. • Improving supervision quality has a greater impact than increasing frequency of supervision alone [31].

### Level of education prior to becoming a CHW

There is some evidence that CHWs with higher levels of formal education prior to becoming CHWs are more effective (for example, in record-keeping, diagnosing childhood illness, and appropriately counseling clients), but more highly educated CHWs may also be more likely to drop out after deployment [24]. One review concluded that completion of primary school should be a minimum educational requirement for entering CHW training to meet the needs of underserved communities far from health centers [35].

### Performance measurement

The reviews included in our study provided very little evidence linking routine supervisory performance appraisal to CHW performance as measured by researchers [15]. However, formal supervisory checklists may increase the efficiency of identifying CHWs who are most in need of further training or supervision [20].

### Logistical support and supplies

Regular provision of logistical support and supplies (such as drugs and educational materials) is essential to maintain CHW program effectiveness, productivity, and respect of CHWs by the community [26, 37]. Lack of supplies is demotivating for CHWs [14, 15, 35, 38]. Table 6 summarizes findings from the review literature on logistics and supplies.

**Table 6:** Summary of findings on logistical support and supplies

Topic	Summary of findings
Regular supplies enable effectiveness	*Directly:* Equipping CHWs with the medicines (e.g., drug kits) and supplies (e.g., rapid diagnostic tests, job aids such as checklist and patient forms) that they are trained to use and mandated to have enables them to perform their related roles [27, 35, 38]. *Indirectly: *Community trust and respect can be eroded if CHWs experience frequent stock outs or do not have access to the supplies needed to perform their role [35, 38].
Need for travel support in remote areas	• Travel can be a barrier to effectiveness as CHWs are dependent on road infrastructure and transportation options (e.g., availability of busses); bicycles or a transportation allowance can support CHW access in remote areas [15].
mHealth tools are being explored	• mHealth (mobile technology: phones, personal digital assistants) is being explored as a tool to support CHW work through assisting with diagnostics and enabling communication, reminders, and reporting between the periphery with the center [15, 44, 101, 102].
Low-tech job aids support CHW activities	• Counting beads can be designed to support assessment of rapid breathing [78]. • Treatment cards that remind CHWs how to prescribe drugs [15] and pictorial instructions for rapid diagnostic tests for malaria [61] can improve adherence to guidelines. • Checklists and standard record forms are considered “best practice” for some HIV CHW programs [53].

### Remuneration and incentives

Monetary remuneration (such as salaries, financial incentives, or income from selling commodities) and non-monetary incentives (such as respect, trust, recognition, and opportunities for personal growth, learning, and career advancement) are important motivators for CHWs [15, 19, 23, 33, 39]. In Kok et al.’s [15] review on intervention design factors that influence CHW performance, 25 of the 81 studies with information on incentives reported that CHWs were dissatisfied with their incentives. Satisfaction (or dissatisfaction) with incentives was closely linked to CHW motivation and performance (or lack thereof). Improved financial remuneration can reduce attrition among CHWs in LMICs [23, 40]. CHW rights and the need of CHWs for reliable financial remuneration were discussed in only one review, which highlighted Indian CHWs’ consistent (and unmet) demand for salaried positions [41]. Table 7 summarizes findings from the review literature on remuneration and incentives.

**Table 7:** Summary findings on remuneration and incentives

Topic	Summary of findings
Financial incentives	Financial incentives increased motivation: one study in Kok et al.’s review found that CHWs getting financial incentives performed better than CHWs receiving in-kind incentives [15]. However, performance-based incentives focus CHW efforts toward remunerated tasks [15].
Other incentives	Other important incentives are community respect, trust, and recognition (discussed in “Community embeddedness”); personal growth and learning; and access to career progression and other future opportunities [15].
CHW rights	Performance-based incentives, linked to CHWs’ volunteer status and flexible tasks and timings, do not provide financial security and ultimately impede CHW rights [41].

### Deployment

There is no simple formula for determining the optimal size of a CHW’s catchment population. Instead, decisions about catchment area population should be based on a variety of context-specific considerations: frequency of contact required; nature of the services provided; expected weekly time commitment from the CHW; and local geography (including proximity of households), weather, and transport availability [14, 15, 24]. One review [42] found that for interventions consisting of home visits only, there was no consistent effect of the size of the catchment population and neonatal mortality impact. However, when the interventions involved community mobilization as well, the reduction in neonatal mortality was greater when the catchment population for the CHW was smaller. Another related finding was that a high workload can lead to CHW demotivation [23].

### Community embeddedness

Fourteen reviews highlighted aspects of community embeddedness as important enablers of CHW program success [14, 15, 19, 23, 34, 35, 37, 40, 43–48]. CHWs are embedded in communities when community members trust and respect them and feel a sense of ownership over the program, such as can be achieved by giving communities a role in CHW selection and definition of CHW activities [19]. The community’s acceptance of CHWs and their sense that the CHW program is locally appropriate and “owned” is associated with CHW retention, motivation, performance, accountability, and support, and ultimately with the acceptability and uptake of CHWs’ health-related work [14, 15, 19, 23, 38, 40, 47, 49]. Locally trusted CHWs can serve as an effective link between health facilities, health workers, and communities [50], and CHWs who are embedded in their communities can provide services to difficult-to-reach populations [20, 40]. However, CHW embeddedness can lead to CHWs being caught in tensions between the community and the health system as well as between social and biomedical issues [51]. Table 8 summarizes findings from the review literature on community embeddedness.

**Table 8:** Summary findings on community embeddedness

Topic	Summary of findings
Of central importance	Community embeddedness is associated with CHW retention, motivation, performance, accountability, support, and ultimately the acceptability and uptake of CHWs’ health-related work [14, 15, 19, 23, 34, 35, 37, 40, 43–48, 107].
Mechanisms to foster community embeddedness	Community embeddedness can be fostered through [15, 19, 48]: • Community members being involved in CHW selection and selecting a locally admired and trusted person • Community having a clear understanding of and reasonable expectations for their CHW • Community monitoring of CHWs • Community ownership of the CHW program • Community involvement in selection of activities and priority-setting of CHW work • Health system backs up the CHWs with supervision, supplies and support, which in turn helps to maintain community trust in CHWs

### Cost-effectiveness

Research from LMICs has found that shifting aspects of HIV care from higher-level health workers to CHWs is cost-effective [50, 52, 53]. There is some evidence of cost-effectiveness for community case management of malaria by CHWs compared to standard malaria treatment at a health facility [21, 33], for the provision of mental health care by CHWs in LMICs [54], and for the delivery of multiple primary health care interventions [55]. However, one review noted that costing methods varied across studies, making it difficult to generate clear conclusions. The same review also noted that the opportunity costs borne by CHWs for volunteering their time were inadequately accounted for [33]. Table 9 summarizes findings from the review literature on cost-effectiveness.

**Table 9:** Summary findings on cost-effectiveness

Topic	Summary of findings
Evidence that CHWs are cost-effective	• CHWs in LMICs are cost effective when compared to standard care for tuberculosis; weaker evidence of cost effectiveness is present for other areas (malaria programs and reproductive, maternal, newborn, and child health) [55]. • Task shifting to CHWs from higher-level staff for HIV care in LMICs is cost effective [50, 52, 53]. • There is a cost savings of 24% when CHWs collect data using personal digital assistants compared to when they use traditional manual methods of data collection and transmission [44]. • Women’s groups (which were almost always facilitated by CHWs) practicing participatory learning and action to improve maternal and newborn health in LMICs were cost-effective as defined by WHO standards [105]. • Pediatric asthma care in HICs by CHWs may be cost-effective [56, 57]. • Diabetes care in HICs by CHWs could save US$2000 annually per Medicaid participant (according to one study) [125]; yield a return on investment of $2.28 per dollar invested (one study) [125], and reduce inappropriate health care utilization [100]. • Community case management of malaria by CHWs using rapic diagnostic tests is cost-effective in areas with low-to-medium prevalence [21]. • Potential cost savings are present by using CHWs for mental, neurological, and substance-abuse disorders in LMICs [54].
Some cost-effectiveness analyses found no evidence	• The evidence regarding the cost effectiveness of vaccination promotion by CHWs in LMICs is inconclusive [59]. • There are no studies of the cost effectiveness of CHWs for the support of HIC populations with vascular disease [60]. • There are insufficient data to assess the cost-effectiveness of CHWs in the USA underserved groups compared to other types of community health interventions [25].

In HICs, interventions delivered by CHWs to reduce triggers for childhood asthma brought cost savings [56, 57]. Another HIC study reported cost savings associated with peer support for breastfeeding [58]. Three reviews found inconclusive or no evidence on cost-effectiveness: vaccination promotion in LMICs [59], control of vascular diseases in HICs [60], and outreach to underserved groups in the USA [25].

### Integration into health systems

The integration of CHW programs into the health system is reported in many reviews to be a key enabler [14, 15, 17, 19, 23, 24, 26, 32, 34, 35, 38, 61]. Pallas et al. [23] highlight that the integration of CHW programs into the agendas of the ministry of health, NGOs, and international donors can strengthen CHW programs and can also help bolster programs in times of political upheaval, loss of external donor funding, and reduced prioritization by the ministry of health. Integration that fosters respectful collaboration and communication between CHWs and higher-level staff can enable the health system to benefit from the unique, practical knowledge that CHWs have and can support CHW retention; this integration can enhance the acceptability and credibility of CHW programs [14, 15, 19, 24, 38, 44]. Table 10 summarizes findings from the review literature on health system integration.

**Table 10:** Summary findings on health system integration

Topic	Summary of findings
Integration with the health system is essential for having strong programs	• Integration and cooperation with the broader health system and existing healthcare providers was the most frequently cited enabling factor for CHW programs in one review [23] and discussed as a vital enabler in many other reviews [15, 19, 20, 35, 38, 47, 48]. • The lack of a national CHW policy has been linked to: • Inadequate support and recognition for CHWs, which limits their ability to function effectively in the community; • Issues around role definition (e.g., whether CHWs should treat illnesses and prescribe medications) [24].
Scaling up and integrating CHW programs with health systems has risks and pitfalls	• A national CHW policy by itself is insufficient; the health system needs to be equipped to supervise, support, and incentivize CHWs [24]. • Scaled-up, integrated CHW programs are often less effective than small, NGO CHW programs because insufficient attention is given to maintaining the quality of the training, supervision, and motivation of CHWs in scaled-up programs [42]. • Integration with a dysfunctional health system can erode CHW programs [35].
Integration with health systems should be built on collaborative, respectful relationships	• Integration must foster respectful collaboration and trust between CHWs and the health system, and it can be facilitated by role clarity and effective two-way communication [15] (potentially supported by mHealth [44]). • The less hierarchical and the more collaborative are relationships between CHWs and the health system, the greater is the likelihood of benefitting from the unique, practical knowledge that CHWs have [19]; moreover, these collaborative relationships can support CHW retention [19, 24, 38]. • Engagement with stakeholders (policymakers, government officials, civil society and communities) fosters integration by enhancing acceptability and credibility of the CHW program [38].

## Discussion

CHWs perform many roles in high-, middle-, and low-income country health systems and contribute to improving a range of health outcomes. However, their capacity is directly contingent on the support they receive from the health system. This review of reviews identifies a number of broad health system supports that optimize CHW programs and can be considered in light of context-specific factors to support health policy decision-making. It finds that CHW tasks should be clearly defined and should require a time commitment appropriate to the incentives/remuneration and support provided. Training should seek to impart both technical competency and socially oriented skills such as communication and counseling, including on confidentiality. Training appears to be more effective in imparting competencies by integrating hands-on practical components rather than just providing classroom learning and should be closely linked to ongoing supportive (rather than punitive or bureaucratic) supervision. Regular provision of supplies, such as medicines, communication tools and teaching aids, and transportation support, is essential for maintaining CHW program effectiveness. The review finds strong support for ensuring community embeddedness, as this is associated with CHW retention, motivation, performance, accountability, and support -- and ultimately affects the acceptability and uptake of CHWs’ health-related work. Linking CHWs to a supportive and functioning referral facility is often vital to CHW program effectiveness. Furthermore, programs must develop appropriate financial and non-financial incentives that take into account a range of factors, including the health system’s resource availability, CHW needs, rights, and expectations, and the tasks and time commitments required. The size of a CHW’s catchment population should be determined in response to the local reality, including population density, travel required, and workload.

As many countries are in the process of implementing new national CHW programs or strengthening current ones, the evidence synthesized in this review can help optimize these efforts. Ultimately, CHW programs are highly context specific. There are no standard blueprints that can be used to design and implement a CHW program. When developing programs, decisions must be made based on national, sub-national, district, and local realities.

This review also enabled the identification of several gaps in the review evidence. Relatively more (and higher quality) evidence is available on the effectiveness of CHWs in delivering specific health interventions than on effective approaches and cross-cutting strategies to integrate and support CBPs in health systems and optimize their performance [62]. There is little discussion in the review literature on the rights and needs of CHWs (with notable exceptions [36, 41]), on effective approaches to training and supervision, on CHWs as community change agents, as multisectorial actors, and on the influence of health system decentralization, social accountability, and governance.

Effectively addressing population needs for Universal Health Coverage with realistically available resources requires harnessing opportunities from the education and deployment of CHWs as members of inter-professional primary health care teams [1]. Countries should develop policies and mechanisms to integrate CHWs with the health system so as to enable these cadres to benefit from health system support and to enable the health system to achieve optimal benefit from CHWs [63]. Health system integration should foster respectful communication and collaboration between CHWs and other health system actors.

Integration of CHWs with health systems requires their inclusion into public policies, including those related to national human resources for health planning, governance, legal frameworks, and financing for health services. The requisite inputs of human and financial resources should be factored in at planning and budgeting stages and should be reflected in national health workforce and health sector strategies.

Policy dialogue about creating a strong role for CHWs in health systems must also address human and labor rights issues surrounding the CHW workforce [64], the favorable consequences of employment of large numbers of CHWs for economic growth and social development [64–66], as well as for achieving the Sustainable Development Goals [67].

This review faced some limitations. In including a range of study types—meta-analyses, systematic reviews, and non-systematic reviews (e.g., scoping, narrative, realist reviews)—and synthesizing findings across a broad range of issues and contexts, it was not possible to assess the overall risk of bias in the findings or to systematically account for the variable quality of the included reviews. Furthermore, presenting findings synthesized from a range of reviews necessitated a high level of abstraction and limited our capacity to present specific and important details and findings from individual studies. We encourage readers to examine AMSTAR quality scores and strength of evidence assessments in Additional file 3 for specific articles and to return to the source materials referenced for more information on topics of interest. Our definition of CHWs may not match definitions used by other teams, leading to inclusions and exclusions that may not fit the needs of all readers. In including research from high-, middle-, and low-income countries, some findings from drastically different settings may be difficult to transfer and apply. In addition, we focused only on academic, peer-reviewed literature, likely missing out on important findings from the gray literature.

## Conclusion

The findings from this review can be adapted to national contexts, where the available resources to support CHW programs are highly variable. Developing and strengthening CHW programs will involve taking into account existing evidence of CHW program effectiveness, weighing options in light of a country’s existing primary health care system and needs, making informed decisions involving all stakeholders, designing and implementing the best program possible, and then adjusting course on the basis of experience, monitoring and evaluation, and findings from rigorous implementation research. Future progress in improving CHW programs will depend not only on synthesizing existing evidence but also on supporting and funding research to continually advance the contextualized evidence on how to design and implement CHW programs to maximize effectiveness [68]. CHWs can play a key role in strengthening health systems to provide universal, comprehensive, and people-centered care that is equitable, culturally appropriate, and economically feasible [1].

## Additional files

Additional file 1: PUBMED search strategy. (DOCX 168 kb)

Additional file 2: AMSTAR quality appraisal. (DOCX 37 kb)

Additional file 3:  Searchable Excel database summary of all reviews. (XLSX 351 kb)

Additional file 4: Summary information by topic from the 122 reviews. (DOCX 129 kb)

Additional file 5: Included and excluded articles. (DOCX 55 kb)

## Abbreviations

AMSTAR: Assessing the Methodological Quality of Systematic Reviews
CHW: Community-based health worker
HIC: High-income country
HIV: Human immunodeficiency virus
LMIC: Low- and middle-income country
NCD: Non-communicable disease
TBA: Traditional birth attendant
WHO: World Health Organization
