# Supplementary material for: What do we know about community-based health worker programs? A systematic review of existing reviews on community health workers
Source: Hum Resour Health. 2018 Aug 16;16:39. doi: 10.1186/s12960-018-0304-x (PMC6097220; doi:10.1186/s12960-018-0304-x)
Supplement: Supplementary file 1 — PUBMED search strategy. (DOCX 168 kb) [file 12960_2018_304_MOESM1_ESM.docx]

**Web Appendix 1. PUBMED search strategy**

**Concept 1: PubMed systematic review filter**

(systematic review [ti] OR meta-analysis [pt] OR meta-analysis [ti] OR systematic literature review [ti] OR this systematic review [tw] OR pooling project [tw] OR (systematic review [tiab] AND review [pt]) OR meta synthesis [ti] OR meta synthesis [ti] OR integrative review [tw] OR integrative research review [tw] OR rapid review [tw] OR consensus development conference [pt] OR practice guideline [pt] OR drug class reviews [ti] OR cochrane database syst rev [ta] OR acp journal club [ta] OR health technol assess [ta] OR evid rep technol assess summ [ta] OR jbi database system rev implement rep [ta]) OR (clinical guideline [tw] AND management [tw]) OR ((evidence based[ti] OR evidence-based medicine [mh] OR best practice* [ti] OR evidence synthesis [tiab]) AND (review [pt] OR diseases category[mh] OR behavior and behavior mechanisms [mh] OR therapeutics [mh] OR evaluation studies[pt] OR validation studies[pt] OR guideline [pt] OR pmcbook)) OR ((systematic [tw] OR systematically [tw] OR critical [tiab] OR (study selection [tw]) OR (predetermined [tw] OR inclusion [tw] AND criteri* [tw]) OR exclusion criteri* [tw] OR main outcome measures [tw] OR standard of care [tw] OR standards of care [tw]) AND (survey [tiab] OR surveys [tiab] OR overview* [tw] OR review [tiab] OR reviews [tiab] OR search* [tw] OR handsearch [tw] OR analysis [ti] OR critique [tiab] OR appraisal [tw] OR (reduction [tw]AND (risk [mh] OR risk [tw]) AND (death OR recurrence))) AND (literature [tiab] OR articles [tiab] OR publications [tiab] OR publication [tiab] OR bibliography [tiab] OR bibliographies [tiab] OR published [tiab] OR pooled data [tw] OR unpublished [tw] OR citation [tw] OR citations [tw] OR database [tiab] OR internet [tiab] OR textbooks [tiab] OR references [tw] OR scales [tw] OR papers [tw] OR datasets [tw] OR trials [tiab] OR meta-analy* [tw] OR (clinical [tiab] AND studies [tiab]) OR treatment outcome [mh] OR treatment outcome [tw] OR pmcbook)) NOT (letter [pt] OR newspaper article [pt])

**Concept 2: CHWs**

"Community Health Workers"[Mesh] OR "Community Health Nursing"[Mesh] OR "health auxiliary"[tw] OR "frontline health workers"[tw] OR "frontline health worker"[tw] OR "midwife"[tw] OR "Midwifery"[tiab] OR "midwives"[tw] OR "Birth Attendant"[tw] OR "Midwives"[tw] OR "outreach worker"[tw] OR "outreach workers"[tw] OR "lay health worker"[tw] OR "lay health workers"[tw] OR "promotora"[tw] OR "promotoras"[tw] OR "village health worker" OR "village health workers"[tw] OR "volunteer health worker"[tw] OR "volunteer health workers"[tw] OR "voluntary health workers"[tw] OR "voluntary health worker"[tw] OR "community health agent"[tw] OR "community health agents"[tw] OR "health promoter"[tw] OR "health promoters"[tw] OR "community health worker"[tw] OR "community health workers"[tw] OR "community health aide"[tw] OR "community health aides"[tw] OR "community health nursing"[tw] OR "community health nurses"[tw] OR "community health nurse"[tw] OR "community health officers"[tw] OR "community health officer"[tw] OR "community health volunteer"[tw] OR "community health volunteers"[tw] OR "community health distributors"[tw] OR "community health distributor"[tw] OR "community health surveyors"[tw] OR "community health surveyor"[tw] OR "community health assistants"[tw] OR "community health assistant"[tw] OR "community health promoters"[tw] OR "community health promoters"[tw] OR "community IMCI"[tw] OR "community volunteer"[tw] OR "community volunteers"[tw] OR "health extension workers"[tw] OR "health extension worker"[tw] OR "village health volunteer"[tw] OR "village health volunteers"[tw] OR “close-to-community provider”[tw] OR “close-to-community providers”[tw] OR "community-based practitioner”[tw] OR “community-based practitioners”[tw] OR “lady Health worker”[tw] OR “lady Health workers”[tw] OR “barefoot doctor”[tw] OR "Community Practitioners"[tw] OR "Community Practitioner"[tw] OR "community-based practitioners"[tw] OR "community-based practitioner"[tw] OR "promotoras de salud"[tw] OR "agentes de saúde"[tw] OR "rural health auxiliaries"[tw] OR "traditional birth attendants"[tw] OR "traditional birth attendant"[tw] OR "Activista"[tw] OR "Agente comunitario de salud"[tw] OR "Agente comunitário de saúde"[tw] OR "Anganwadi"[tw] OR "Animatrice"[tw] OR "Barangay health worker"[tw] OR "Barangay health workers"[tw] OR "Basic health worker"[tw] OR "Basic health workers"[tw] OR "Brigadista"[tw] OR "Colaborador voluntario"[tw] OR "Community drug distributor"[tw] OR "Community drug distributors"[tw] OR "Community health agent"[tw] OR "Community health agents"[tw] OR "Community health promoter"[tw] OR "Community health promoters"[tw] OR "Community health representative"[tw] OR "Community health representatives"[tw] OR "Community health volunteer"[tw] OR "Community health volunteers"[tw] OR "Community resource person"[tw] OR "Female multipurpose health worker"[tw] OR "Female multipurpose health worker"[tw] OR "Health promoter"[tw] OR "Health promoters"[tw] OR "Kader"[tw] OR "Monitora"[tw] OR "Mother coordinator"[tw] OR "Outreach educator"[tw] OR "Outreach educators"[tw] OR "Promotora"[tw] OR "Shastho shebika"[tw] OR "Shastho karmis"[tw] OR "Sevika"[tw] OR "Village health helper"[tw] OR "Village drug-kit manager"[tw] OR "Accompagnateur"[tw] OR "Accredited Social Health Activist"[tw] OR "Animator"[tw] OR "ASHA"[tw] OR "Auxiliary Nurse"[tw] OR "Auxiliary Nurse-midwife"[tw] OR "Bridge-to-Health Team"[tw] OR "Behvarz"[tw] OR "Care Group"[tw] OR "Care Groups"[tw] OR "Care Group Volunteer"[tw] OR "Care Group Volunteers"[tw] OR "Community Case Management Worker"[tw] OR "Community Case Management Workers"[tw] OR "Community Health Agent"[tw] OR "Community Health Agents"[tw] OR "Community Health Care Provider"[tw] OR "Community Health Care Providers"[tw] OR "Community HealthCare Provider"[tw] OR "Community HealthCare Providers"[tw] OR "Community Health Extension Worker"[tw] OR "Community Health Extension Workers"[tw] OR "Community Health Officer"[tw] OR "Community Health Officers"[tw] OR "Community Surveillance Volunteer"[tw] OR "Community Surveillance Volunteers"[tw] OR "Family Health Worker"[tw] OR "Family Health Workers"[tw] OR "Family Planning Agent"[tw] OR "Family Planning Agents"[tw] OR "Family Welfare Assistant"[tw] OR "Family Welfare Assistants"[tw] OR "Female Community Health Volunteer"[tw] OR "Female Community Health Volunteers"[tw] OR "Health Agent"[tw] OR "Health Agents"[tw] OR "Health Assistant"[tw] OR "Health Assistants"[tw] OR "Health Extension Worker"[tw] OR "Health Extension Workers"[tw] OR "Health Surveillance Assistant"[tw] OR "Health Surveillance Assistants"[tw] OR "Kader"[tw] OR "Lead Mother"[tw] OR "Malaria Agent"[tw] OR "Malaria Agents"[tw] OR "Maternal and Child Health Worker"[tw] OR "Maternal and Child Health Workers"[tw] OR "Mobile Clinic Team"[tw] OR "Mobile Clinic Teams"[tw] OR "Nutrition Agent"[tw] OR "Nutrition Agents"[tw] OR "Nutrition Counselor"[tw] OR "Nutrition Counselors"[tw] OR "Peer Educator"[tw] OR "Peer Educators"[tw] OR "Shasthya Shebika"[tw] OR "Socorrista"[tw]
