# Supplementary material for: What do we know about community-based health worker programs? A systematic review of existing reviews on community health workers
Source: Hum Resour Health. 2018 Aug 16;16:39. doi: 10.1186/s12960-018-0304-x (PMC6097220; doi:10.1186/s12960-018-0304-x)
Supplement: Supplementary file 2 — AMSTAR quality appraisal. (DOCX 37 kb) [file 12960_2018_304_MOESM2_ESM.docx]

**Web Appendix 2. AMSTAR Quality Appraisal**

**Adaptation and application of the**

**AMSTAR quality appraisal**

Two authors (SWB and MG) rated each systematic review using AMSTAR criteria (Shea et al., 2007), which is a validated quality review including 11 items. Reviews that we included but were not systematic were not rated with AMSTAR. These same two authors each rated the same 10 systematic reviews using the AMSTAR quality rating, discussed their findings together, and then came to a consensus on how to apply the AMSTAR criteria before rating the remainder of the systematic reviews.

All review articles that were systematic reviews were assessed for quality using the AMSTAR criteria. We classified reviews as non-systematic if the authors explained that they had not carried out a systematic approach or if they had no clear predefined methodology for obtaining articles and analyzing/synthesizing the findings.

Although AMSTAR was designed to rate the quality of systematic reviews of randomized controlled trials, most of the included systematic reviews that met our selection criteria were composed of studies using non-experimental methodologies. We found that for the systematic reviews selected for our study, some of the AMSTAR criteria were not readily applicable or subject to interpretation. Thus, a consensus was reached among the reviewers on how to apply some of the checklist items as follows:

- AMSTAR item 1: Was *a priori* design provided?
  - Articles received a “yes” rating if the research question and inclusion criteria were established before the conduct of the review
- AMSTAR item 2: Was there duplicate study selection and data extraction?
  - Articles in this review were given a “yes” rating only if there was duplicate review at both screening and data extraction.
- AMSTAR item 3: Was a comprehensive literature search performed?
  - Articles in this review were given a “yes” rating only if at least 2 databases were searched.
- AMSTAR item 4: Was the status of publication (i.e. grey literature) used as an inclusion criterion?
  - Articles were given a “yes” rating if the authors searched grey literature.
- AMSTAR item 5: Was a list of studies (included and excluded) provided?
  - Relaxed interpretation: Articles were given a “yes” rating only if the studies included in the review were listed (it was not necessary to provide a list of excluded studies in order to receive a “yes”). We decided to create this relaxed criteria because we noted that requiring a list of excluded articles was highly restrictive and met by very few reviews.
  - Strict interpretation: Articles were given a “yes” rating only if included and excluded articles were listed.
- AMSTAR item 6: Were the characteristics of the included studies provided?
  - Articles received a “yes” if data on the participants, interventions and outcomes from the original studies was presented an aggregated form such as a table.
- AMSTAR item 7: Was the scientific quality of the included studies assessed and documented?
  - Articles received a “yes” if authors assessed the quality of the studies included in their review
- AMSTAR item 8: Was the scientific quality of the included studies used appropriately in formulating conclusions?
  - Articles received a “yes” if the authors considered the results of their quality assessment in the analysis and the conclusions of the review.
- AMSTAR item 9: Were the methods used to combine the findings of studies appropriate?
  - We decided to apply this specifically to meta-analyses and meta-synthesis; if no meta-analysis or meta-synthesis was conducted, we gave a rating of “not applicable.”
- AMSTAR item 10: Was the likelihood of publication bias assessed?
  - Relaxed interpretation: No funnel plot/quantitative summary measure was needed to receive a “yes.” If the authors acknowledged possibilities of publication bias and how that might influence their results, we assigned a “yes.” We decided to create this relaxed criteria because many experts consider funnel plots to not be useful for assessing publication bias, some of the reviews were qualitative or mixed, and funnel plots are not widely used.
  - Strict interpretation: A funnel plot/quantitative summary measure was needed to receive a “yes”. Two articles that discussed not being able to construct a funnel plot due to insufficient data (Kew, Carr, & Crossingham, 2016; Oyo-Ita et al., 2016) were still given a “yes” assessment because their non-use of a funnel plot was appropriate.
  - For qualitative meta-synthesis reviews (Glenton et al., 2013; Hall et al., 2017; Ma et al., 2016; Tso et al., 2016), we considered an assessment of bias to be not applicable.
- AMSTAR item 11: Was the conflict of interest included?
  - As long as the authors listed their own conflicts of interest and funding sources, we assigned a “yes” to this item; there was no need to list conflicts/sources for each article included in the respective review in order to assign a “yes” to this item.

After the initial 10 reviews were completed in duplicate and a consensus obtained on how to proceed, the remaining systematic reviews were rated by one reviewer; a random sample of 10% of systematic reviews were rated in duplicate to check agreement. Disagreements were limited and resolved through discussion.

Web Appendix 1, Table 1 presents the summary of quality for the systematic reviews by AMSTAR item. Please note that the ratings for each article are available in Web Appendix 1.

| **Web Appendix 1, Table 1. Quality of systematic reviews by AMSTAR item** | | | |
| --- | --- | --- | --- |
| **AMSTAR quality item** | **Reviews meeting criteria (n)** | **Reviews eligible (N)** | **(n/N) %** |
| Item 1. Review has a priori design | 75 | 75 | 75/75 (100%) |
| Item 2. Duplicate data screening and data extraction | 47 | 75 | 47/75 (63%) |
| Item 3. Literature search in at least two databases, with years and databases listed | 73 | 75 | 73/75 (97%) |
| Item 4. Grey literature included | 39 | 75 | 39/75 (52%) |
| Item 5a (relaxed). Included articles listed | 71 | 75 | 71/75 (95%) |
| Item 5b (strict). Included and excluded articles listed | 17 | 75 | 17/75 (23%) |
| Item 6. Characteristics of included articles listed (e.g., information on participants, interventions, and outcome) | 70 | 75 | 70/75 (93%) |
| Item 7. Quality assessment | 65 | 75 | 65/75 (87%) |
| Item 8. Quality review incorporated into the conclusion | 58 | 64 | 58/64 (91%) |
| Item 9. Appropriate use of meta-analysis | 34 | 34 | 34/34 (100%) |
| Item 10a (relaxed). Publication bias discussed | 43 | 71 | 42/70 (61%) |
| Item 10b (strict). Publication bias assessed through funnel plot or other quantitative summary measure | 12 | 71 | 12/71 (17%) |
| Item 11. Conflict of interest or funding information included | 71 | 75 | 71/75 (95%) |
