# Supplementary material for: What do we know about community-based health worker programs? A systematic review of existing reviews on community health workers
Source: Hum Resour Health. 2018 Aug 16;16:39. doi: 10.1186/s12960-018-0304-x (PMC6097220; doi:10.1186/s12960-018-0304-x)
Supplement: Supplementary file 4 — Summary information by topic from the 122 reviews. (DOCX 129 kb) [file 12960_2018_304_MOESM4_ESM.docx]

**Web Appendix 4: Summary Information by Topic from the 122 Reviews**

**Index of tables**

Table 1. Health system-related HIC reviews 2

Table 2. Underserved population-related HIC reviews 3

Table 3. Maternal health and breastfeeding HIC reviews 4

Table 4. Non-communicable disease HIC reviews 5

Table 5. Other HIC reviews 8

Table 6. PHC LMIC reviews 9

Table 7. Health system-related LMIC reviews 12

Table 8. CBP rights and wellbeing-related LMIC reviews 13

Table 9. Maternal and child health LMIC reviews 14

Table 10. Non-communicable disease LMIC reviews 23

Table 11. Infectious disease LMIC reviews 24

Table 12. Other LMIC reviews 29

Community-based practitioner roles, capacities and health outcomes as reported in reviews from high-income countries (HICs)

Four reviews examined CBPs in HIC settings addressing multiple health issues in the health system (Table 1).

| Table 1. Health system-related HIC reviews | | | |
| --- | --- | --- | --- |
| **1^st^ author** | **Date** | **Review type** | **Main outcomes and CBP roles** |
| Dale | 2008 | SR MA | There is some evidence from HICs that peer-support telephone calls can be effective for certain health-related concerns (mammography screening, diet change in post-myocardial infraction patients, breastfeeding continuation, and reducing depressive symptoms in mothers with postnatal depression). |
| Lizarondo | 2010 | NSR | There is consensus in the HIC literature that allied health assistants (AHAs) make a valuable contribution to allied health care. However, ongoing barriers to their effective use persist. AHAs have duties that require contact with patients (such as administration of clinical services, preparation of patients, patient education, and supervision of patients) and duties that do not require direct patient contact (such as administrative and clerical duties). |
| South | 2013 | NSR | In HICs, the features of lay health worker programs can be grouped into four dimensions: intervention, role, professional support/service and the community. More account needs to be taken of the variations among lay health worker programs. The dimensions of lay health worker programs should not be mutually exclusive, as many programs seek to combine various roles in a hybrid fashion, and furthermore roles may evolve over time. Bridging models (where CHWs connect underserved communities to health services) are prominent in studies carried out in North America; however, the roles can also involve peer education and culturally appropriate support. |
| Stanhope | 2013 | SR | Allied health assistants (AHAs) in HICs are being employed in a range of settings, and they appear to be effective in terms of process measures and stakeholder perceptions. AHAs are well-accepted by clients, provide clients with more therapy time (e.g., for speech therapy or physiotherapy), and free up time for health professionals to perform other duties. |

Seven reviews examined CBPs in HIC settings addressing health issues specifically facing underserved populations: five on racial and ethnic health disparities in the US (including one specifically on US Hispanics/Latinos), one on immigrant and ethnic minority adults in the US and Canada, and one on Aboriginal health in Australia (Table 2).

| Table 2. Underserved population-related HIC reviews | | | |
| --- | --- | --- | --- |
| **1^st^ author** | **Date** | **Review type** | **Main outcomes and CBP roles** |
| Abbott | 2017 | SR | Focus on efforts to address social determinants of health through home visiting for racial and ethnic minorities in the US. Of the 39 included studies, 20 involved CHWs alone or in teams conducting community and home visitation interventions. Evidence from experimental studies (e.g. intervention vs. control) found that home visits conducted by CHWs alone or in teams can positively influences a range of health-related behaviors, understandings and attitudes among marginalized people, including: improving parenting knowledge, knowledge of influenza prevention, home safety, self efficacy, pap screening, Hep B testing, knowledge about prostate cancer (but not screening) and mammography attainment; increasing breastfeeding attempts and duration, number of asthma free days, and infant-stimulating home environment scores; reducing depression, urgent care visits, obesity among postpartum teens, unplanned repeat births among adolescents, and psychiatric diagnoses among children. |
| Gibbons | 2007 | SR | Focus on the role of CHWs in addressing racial and ethnic disparities in health status and health care in the US. The available evidence from randomized controlled trials in the US suggests positive benefits may be attributable to the use of CHWs. CHWs in the US conduct home visits, make referrals and lead counseling sessions. They also provide education (e.g., giving colorectal cancer information) and organize activities. |
| Islam | 2015 | NSR | CHW programs for Asian American, and Native Hawaiian, and Pacific Islander communities in the US do not adequately address the ethnic and cultural diversity in these populations. CHW programs should be geographically expanded to reach these populations across the country. CHWs are underutilized in addressing a range of health issues in these communities, including mental health disparities, HIV/ AIDS, and occupational health/injuries. |
| Mercer | 2014 | SR | In Australia, workplace culture and environments impact on the experiences of Aboriginal health workers (who are CBPs) and non-Aboriginal health care providers working in collaborative clinical arrangements. When Aboriginal health workers and non-Aboriginal health care providers are empowered to work in a successful clinical partnership, through an enabling workplace, there is a great benefit for both the health professional and Aboriginal health worker, the Aboriginal community and the health service. |
| Rhodes | 2006 | NSR | Lay health advisors have been used to promote health in US Hispanic/Latino communities for a range of issues, including cancer screening, cardiovascular health, prenatal care, and diabetes management. Roles include providing advice, facilitating referrals, distributing materials, serving as role models, and advocating on behalf of community members. Most LHAs were female, and training ranged from six to 160 hours. Only 14 of the 37 studies identified offered evidence of effectiveness, 12 of which used controls or a comparison group; all showed positive outcomes. |
| Shommu | 2016 | SR | Community health navigators (healthcare workers who support patients to obtain appropriate healthcare) can improve chronic disease management and access to primary health care for screening (e.g., cancer screening or body mass index assessment) for immigrant and ethnic minority adults. The one Canadian study included in the review also reported positive outcome of navigators among immigrant women. While navigator interventions have not been fully explored in Canada, in the US many studies have demonstrated significant improvements in immigrant health outcomes. |
| Viswana-than | 2009 | SR MA | In the US, CHWs can serve as a means of improving outcomes for underserved populations for some health conditions. There is moderate evidence that CHW interventions improve the knowledge of participants on disease prevention and cancer screening, and there is weak evidence that CHW interventions improve knowledge of medication label reading among diabetics. Studies suggested that CHW interventions result in improvements in participant behavior when compared with alternatives such as a community intervention (not further defined), a lower-intensity CHW intervention, and usual care (without a CHW) combined with a pamphlet. The strength of evidence is moderate for the use of bedding encasements for asthma, and low for workplace safety and diabetes mellitus. The evidence for disease prevention, improving the environment for child well-being, planned use of cancer screening tests, and breast self-examination is mixed. CHWs provided interventions in health promotion and disease prevention, injury prevention, maternal and child health, cancer screening and chronic disease management, with varying degrees of effectiveness. |

Three reviews examined CBPs in HIC settings on maternal health issues: two on breastfeeding support and one on maternal mental health (Table 3).

| Table 3. Maternal health and breastfeeding HIC reviews | | | |
| --- | --- | --- | --- |
| **1^st^ author** | **Date** | **Review type** | **Main outcomes and CBP roles** |
| Chapman^[[1]](#footnote-1)^ | 2010 | NSR | The overwhelming majority of evidence from RCTs indicates that peer counselors improve rates of breastfeeding initiation, duration, and exclusivity, and this leads to decreases in the incidence of infant diarrhea and duration of lactational amenorrhea. Breastfeeding peer counseling can be scaled up in both developing and developed countries. |
| Jones | 2014 | SR MA | In HICs, access to the right type of peer support can positively impact women's perinatal mental health. It is important to recognize the risk of isolation and to establish pathways of referral to peer support networks. Practitioners must nurture peer support networks in perinatal care. More research is required to establish the most successful formats/structures of peer support. |
| Kaunonen | 2012 | SR | In HICs, individual support and education were the most common peer interventions for breastfeeding support. 12 of the 15 studies reporting breastfeeding continuation showed peer support to be effective. |

Fifteen reviews examined CBPs in HIC settings addressing non-communicable diseases: five on diabetes, three on cancer, two on pediatric chronic conditions (one general and one specifically about asthma), two on mental health, and one each on hepatitis, hypertension, and vascular disease. In addition, one review (Kew, Carr, & Crossingham, 2016), on asthma, had a global scope but included primarily high-income setting data and is thus also included below (Table 4).

| Table 4. Non-communicable disease HIC reviews | | | |
| --- | --- | --- | --- |
| **1^st^ author** | **Date** | **Review type** | **Main outcomes and CBP roles** |
| **Diabetes** | | | |
| Cherring-ton | 2008 | NSR | US-only: five CHW roles in diabetes management were identified: (1) educator, (2) case manager, (3) role model, (4) program facilitator, (5) advocate. Roles, responsibilities and training varied greatly as did outcomes that were assessed. Future studies are needed regarding how to integrate CHWs into diabetes programs. |
| Hunt | 2011 | SR | US-only: Community health advisor interventions for people with type-2 diabetes were based on providing culturally appropriate care and resolution of health disparities within minority populations in the US. Typically, community health advisors had diabetes themselves. Major roles of community health advisors included working as a supporter, educator, case manager, advocate, and program facilitator. Activities included: coordinating educational programs, conducting educational courses for patients, serving as a link between patients and healthcare professionals, providing counseling, and leading peer-support meetings. The effectiveness of interventions by community health advisors was mixed. Decreases in HbA1C (a marker for diabetes) were noted in several studies, but in some cases the change was not statistically significant. |
| Little | 2014 | SR | Seven out of 12 RCTs on CHW-delivered interventions for Latinos with type 2 diabetes in the US reported statistically significant improvements in glucose levels as measured by HbA1c. The methodological quality was good but there were inconsistencies in the reporting of key information. Future research should (1) report in greater detail CHW background, training, and supervision; (2) examine factors associated with intervention effectiveness; and (3) provide data on cost and cost-effectiveness. CHWs led the intervention either alone, in pairs, or as part of a team (not further specified), with responsibilities including educating, advocating, supporting, and coordinating logistics. CHWs were described as paid staff in six studies. |
| Norris | 2006 | NSR | Diabetes patients were generally satisfied with their contacts with CHWs, and patient knowledge about diabetes increased. Improvements in physiological measures were noted for some interventions, and positive changes in lifestyle and self-care were noted in a number of studies. There were few data on economic outcomes, but several studies demonstrated a decrease in inappropriate health care utilization. CHW roles varied from substantial involvement in patient care to simply providing assistance with implementing education sessions taught by other health professionals. |
| Palmas | 2015 | SR MA | CHW interventions showed a modest reduction in glycaemia (level of HbA1c) compared to usual care. CHW roles included facilitating group classes, contacting patients by telephone, and conducting outreach. The number of CHW-participant encounters varied widely. In terms of cost-effectiveness, one study suggested that a CHW program in diabetes care could save $2,000 annually per Medicaid participant, and one study found that an outreach program for underserved men in Denver, Colorado, could yield a return on investment of $2.28 per dollar invested. |
| **Cancer** | | | |
| Hou | 2015 | NSR | In the US, community and clinic health navigators for cancer screening promotion had similar roles and responsibilities. Most of the navigator interventions increased cancer screening knowledge and behaviors versus usual care or comparison groups. Navigator roles included: reaching out to participants via mail, phone, or face-to-face contact; providing information or hosting educational events; providing counseling; sharing personal testimony; providing logistical support, particularly helping schedule screening; serving as a translator or advocate; and/or gathering information about access to cancer care screenings. |
| Martinez-Donate | 2009 | NSR | In the US., there is limited evidence of effectiveness of CHW interventions to promote breast and cervical cancer screening among Latinas. Most studies used weak methodological designs. Only 5 of the 14 included studies involved an experimental or quasi-experimental design with a comparison group and pre- and post-test measures. Of these five, only two found evidence of effectiveness. |
| Wells | 2011 | SR MA | In the US, CHW interventions are associated with a statistically significant increase in rates of screening mammography. These interventions tend to have stronger effects in specific settings and study designs as well as when participants and CHWs were similar ethnically or racially. |
| **Pediatric chronic disease** | | | |
| Raphael^[[2]](#footnote-2)^ | 2013 | SR | Lay health worker (LHW) interventions in children with chronic conditions may lead to modest improvements in urgent care use, symptoms, and parental psychosocial outcomes. The improved outcomes that were most commonly reported included reduced urgent care use, decreases in symptoms, fewer missed work and school days, and increased parental quality of life. For children with asthma, LHWs decreased rapid breathing episodes per month, activity limitation, and asthma exacerbations per year, and they also increased the number of symptom-free days per year. For type-1 diabetes, LHWs improved glycaemic control and decreased hospitalizations in intervention groups. LHWs interacted with families at the time of home visits or through phone calls or e-mails. The lay health workers in the 11 studies of asthma primarily focused on education about environmental trigger reduction, provision of engineering controls (e.g., air filters and mattress encasements), asthma action plans, medication management, and increasing parental recognition of symptoms. |
| Postma | 2009 | NSR | In the US, CHW-delivered home-based environmental interventions for pediatric asthma were consistently shown to have positive outcomes, including decreased asthma symptoms, daytime activity limitations, and use of emergency and urgent care. CHWs were effective in counseling to improve patient wellbeing and reducing asthma-triggering behaviors as well as in providing social and emotional support. |
| **Mental health** | | | |
| Hoeft | 2017 | SR | In rural settings, there are several existing models for how CHWs can be involved in mental health care delivery, such as home visitation for pregnant women on Medicaid, support for parents of Latino youth with mental health problems, or door-to-door outreach for stress management, substance abuse, domestic violence, depression, and anxiety. In terms of efficacy, a CHW-led project on depression with an immigrant Latina population created a *fotonovela* (a small illustrated storybook), which resulted in improved depression knowledge, improved efficacy to seek treatment, and decreased stigma toward depression treatment. Provider education, supervision, and partnerships with local communities can support task sharing. Challenges, such as confidentiality, are often not addressed in the literature. |
| Wahlbeck | 2017 | NSR | While beneficial mental health effects of housing and active labour-market interventions have been documented in randomized trials, the evidence for interventions at service and community level is considerably weaker, reflecting challenges in assessing effectiveness of complex and context-dependent interventions by traditional effectiveness study designs. Nonetheless, lay community outreach health workers were able to produce beneficial changes in health status measures in many, but not all, studies. In addition, the lack of evidence of effectiveness of many promising higher-level interventions such as community coalitions, health mediators, social prescriptions and debt advice does not mean they are not effective. Although policies can rarely be evaluated in empirical intervention studies, existing studies point towards the positive mental health impacts of many non-health policies, especially so for social, labour market and housing policies. |
| **Chronic viral hepatitis B and C** | | | |
| Zhou | 2016 | SR MA | A range of relatively simple, inexpensive operational interventions can substantially improve engagement and retention along the chronic viral hepatitis care continuum, thereby optimising the implementation of screening, care, and treatment programs. Meta-analysis demonstrates that educational programs for task shifting to culturally appropriate lay health workers are effective in increasing HBV testing uptake. The six studies included in the meta-analysis were graded as moderate quality evidence. Although all lay health worker interventions were conducted among Asian immigrant populations in high-income countries, this particular type of intervention could apply to other settings as well. Training provided for the lay health workers in the six studies was relatively simple and cheap. |
| **Hypertension** | | | |
| Brown-stein | 2007 | SR | In seven of eight randomized control trials from HICs, CBP interventions for people with hypertension (often underserved African Americans) showed significant improvements in blood pressure. Several significant improvements in participants' self-management behaviors were reported (including appointment keeping and adherence to antihypertensive medications), and positive changes were observed in healthcare utilization (e.g., fewer emergency visits, increased proportion of patients having a nurse or physician) and in health systems outcomes (e.g., provider responsiveness to patient needs). CBP roles included: (1) health education on hypertension (including on treatment, emphasizing need for adherence) and lifestyle changes; (2) assisting patients in accessing services (e.g., obtaining health insurance), (3) directly providing services (measuring and monitoring blood pressure), (4) providing social support, and (5) mediating between participants and the healthcare/social service systems. |
| **Vascular disease** | | | |
| Small | 2013 | SR MA | There is modest evidence from HICs that telephone interventions provided by lay workers and peer-support workers for patients with diabetes can improve self-management and levels of glycaemia (HbA1c), but there is no evidence that this intervention improves mental health or quality of life. |
| **Asthma** | | | |
| Kew^[[3]](#footnote-3)^ | 2017 | SR MA | Although weak evidence suggests that lay-led interventions that provide peer support can lead to a small improvement in asthma-related quality of life for adolescents, benefits for asthma control, exacerbations and medication adherence remain unproven. Current evidence is insufficient to reveal whether routine use of lay-led or peer-support programs is beneficial for adolescents receiving asthma care. Ongoing and future research may help to identify target populations for lay-led and peer-support interventions, along with attributes that constitute a successful program. |

One review examined CBPs in HIC settings promoting physical activity and one examined their role in reducing repeated pregnancies and births among adolescents (Table 5).

| Table 5. Other HIC reviews | | | |
| --- | --- | --- | --- |
| **1^st^ author** | **Date** | **Review type** | **Main outcomes and CBP roles** |
| **Physical activity** | | | |
| Costa^[[4]](#footnote-4)^ | 2015 | SR | This review examined whether physical activity promotion in interventions conducted by CHWs has positive outcomes across HICs. The authors found that 16 of the 26 studies (62%) reported positive results for different parameters of physical activity. CHWs primarily focused on health education counseling delivered in populations at risk or diagnosed with chronic non-communicable diseases. The successful interventions were conducted over a period averaging 6.5 months. |
| Maravilla | 2016 | SR MA | Although most included studies (n=9) were either of “strong” or of “moderate” quality, only two of five studies that found a significant reduction in adolescent repeated childbearing exhibited a high level of quality, as the other three failed to adjust results for confounders. Random effects modeling revealed an overall 30% decrease in repeated adolescent births (odds ratio=0.70, confidence interval: 0.49-0.99) among CHW-visited areas relative to non-visited sites. On the other hand, no significant association was detected in terms of repeated pregnancies (odds ratio=0.96, confidence interval: 0.70-1.28). |

Community-based practitioner roles, capacities and health outcomes as reported in reviews from LMICs

In LMICs, 14 reviews examined CBP involvement in various aspects of PHC (Table 6).

| Table 6. PHC LMIC reviews | | | |
| --- | --- | --- | --- |
| **1^st^ author** | **Date** | **Review type** | **Main outcomes and CBP roles** |
| Agarwal | 2015 | NSR | Frontline health workers are able to learn how to use mHealth tools for (1) data collection and reporting, training and decision support, (3) emergency referrals, (4) work planning through alerts and reminders, and improved supervision of and communication among healthcare workers. Two studies suggest that mHealth can improve adherence to treatment algorithms. 27/42 studies involved CBPs (23 CBPs only, 4 of CBPs and FLHWs together). |
| Bornstein | 2008 | NSR | In Brazil, CHWs are mediators between the health system and the community, and between different forms of health-related knowledge. CHWs amplify issues of citizenship in a general form by supporting the health service to become more “permeable” in terms of knowledge sharing (in either direction), and in responding to the needs and demands of the population. CHWs are at once in positions of power and prestige with respect to their community, but also with the responsibility to absorb community pressure (e.g., from those community members who are seeking favourable treatment, access, etc.). CHWs have both a patient-centred and a biomedical orientation, resulting in tension between different approaches and knowledge bases. Two principal dimensions are discussed in the literature on CHWs – the technical and the political. CHWs are defined as a *sui generis* worker, completely unique, difficult to define, but with an important role that is increasingly important in the healthcare field. Although CHW training is well-documented technically at the MoH level in Brazil, there are diverse ways to implement this in practice, and much variation in CHW training exist around the country. |
| Bosch-Capblanch | 2008 | SR | Supervision is widely recommended, but is a complex and expensive intervention, and implemented in different ways. Supervision interventions generally have shown small positive effects on frontline health worker performance. However, the quality of the studies is mixed and outcomes varied between studies. Three out of 12 effectiveness studies included CBPs. |
| Bosch-Capblanch | 2011 | SR | It is uncertain whether supervision has a substantive positive effect on the quality of primary health care. The long-term effectiveness of supervision is unknown. Of the 9 studies included in the review, only two involved supervision of CBPs. One was an RCT of five day refresher training and supportive monthly supervision visits versus standard supervision (Ayele 1993); the CBPs who received the intervention showed significant improvements in “function status” at six months follow up. The other was an RCT wherein the intervention group received fewer (quarterly) supervisory visits while the control received standard monthly visits (Foreit 1984); there were no significant differences between the intervention and control. |
| Braun | 2013 | NSR | CHWs are using mHealth to collect field-based health data, receive alerts and reminders, facilitate health education sessions, and conduct person-to-person communication. Programmatic efforts using mHealth focus on improving adherence to standards and guidelines, community education and training, and programmatic leadership and management practices. |
| Ehiri | 2014 | SR | There is some evidence, but of weak quality, that deploying lay refugees/internally displaced persons to provide basic health services to other women, children, and families in camps can improve health (as defined by increased service coverage, increased knowledge about disease symptoms and prevention, increased uptake of treatment and protective behaviors, and improved access to reproductive health information). |
| Fulton | 2011 | NSR | Task shifting is an important strategy for addressing needs-based shortages and skill-mix imbalances. Task shifting to CHWs can produce adverse effects. The quality of care provided by CHWs can be suboptimal if CHWs are given complex tasks. Quality and safety concerns may arise, as well as professional and institutional resistance. Sustaining motivation and performance may be a challenge. More research is needed regarding the benefits of tasking shifting from the perspective of comparing outcomes with what would have been the outcomes if no task shifting had occurred. |
| Hill | 2014 | SR | Evidence suggests that improving supervision quality has a greater impact than increasing frequency of supervision alone. Supportive supervision packages, community monitoring of the work of CHWs, and quality improvement/problem-solving approaches show the most promise; however, evaluation of all strategies was weak. |
| Jaskiewicz | 2012 | NSR | The four essential elements of the work environment that affect CHW productivity are: workload, supportive supervision, supplies and equipment, and respect from the community and the health system. There is no known ideal work environment, nor is there a known maximum number or mix of CHW job tasks that will ensure the highest level of CHW productivity. Success is more likely when CHWs have a clear job description, a limited number of tasks, standardized protocols, and job aids that match their training. |
| Källander | 2013 | NSR | Innovative mHealth applications for CHWs include the use of mobile phones as job aides, clinical decision support tools, as well as channels for data submission and instant feedback on performance. The most commonly documented use of mHealth was 1-way text-message and phone reminders to encourage follow-up appointments, healthy behaviors, and data gathering. Although there is vast documentation of project process evaluations, there are few studies demonstrating an impact on clinical outcomes attributable to mHealth interventions. There is also a lack of mHealth applications and services operating at scale in LMICs. |
| Kok, Kane, et al. | 2015 | SR | The review identifies five key contextual factors that influence the performance of CHWs. (1) Community and socio-cultural factors: social and cultural norms, values, practices and beliefs, gender roles and norms, disease-related stigma, safety and security, and education and knowledge level of the CHW beneficiaries. (2) Economic factors: the influence of poverty on one’s willingness to become a CHW, on the health-care seeking behavior of beneficiaries; and on the generalized stress experienced by CHWs and communities. (3) Environmental factors: geography, distance to households, and climate (such as flooding). (4) Health system policies, such as human resources policy, CHW policy, CHW-related legislation, and political commitment. (5) Health system conditions, such as the functionality of health system, the availability of higher-level staff, the decision-making structure, the costs of health services, and the governance/coordination structure. |
| McCollum | 2016 | SR | CHW programs were found to promote equity of healthcare access and utilisation by reducing inequities relating to place of residence, gender, education and socio-economic position and supporting more equitable uptake of referrals. There was no clear evidence for equitable quality of services provided by CHWs and limited information regarding the role of the CHW in generating community empowerment to respond to social determinants of health. However, if CHW programs are not well planned some of the barriers faced by clients at health facility level can replicate at community level. |
| Vaughan | 2015 | NSR | Existing evidence suggests that, compared with standard care, using CHWs in health programs can be a cost-effective intervention in LMICs, particularly for tuberculosis, but also – although the evidence is weaker – in other areas such as reproductive, maternal, newborn and child health and malaria. |
| WHO/ GHWA | 2010 | NSR | Almost all of the intervention studies involving CHWs showed a significant impact on reducing maternal, perinatal and neonatal mortality and on improving perinatal and postpartum service utilization indicators. CHWs provide a range of services, from safe deliveries to counseling and preventive health education to the treatment and rehabilitation of people suffering from common mental health problems. The services offered by CHWs have helped in the decline of maternal and child mortality rates and have decreased the incidence of TB and malaria. |

Seven reviews examined CBPs in LMIC health systems, addressing how best to scale up and sustain CHW programs and the factors that influence CHW program integration into the health system (Table 7).

| Table 7. Health system-related LMIC reviews | | | |
| --- | --- | --- | --- |
| **1^st^ author** | **Date** | **Review type** | **Main outcomes and CBP roles** |
| Baatiema | 2016 | NSR | In Ghana, CHWs have played critical roles in improving health service delivery and outcomes. Examples include their contribution to guinea worm eradication, expanded immunization coverage, maternal and child health, and HIV/AIDS treatment and management. However, these achievements notwithstanding, CHWs face challenges which prevent them from being optimally productive, including capacity problems, neglect by the healthcare system, high attrition rates and inadequate supervision. There is a policy deficit regarding CBPs in Ghana. There is no national framework to guide CBPs, no formal integration into the health system, and a dearth of logistical support, capacity development, career opportunity, and financial security. |
| Kok, Dieleman, et al. | 2015 | SR | CHW performance can, at least in certain settings, be enhanced through a mix of financial and non-financial incentives, frequent supervision, continuous training, and the embedment of CHWs in community and health systems. While supervision and training were often mentioned as facilitating factors, few studies tested which approach worked best or how these were best implemented. Clearly defined CHW roles, clear processes for communication among different levels of the health system, and providing incentives in a predictable way could also strengthen CHW performance. |
| Loures | 2010 | SR | In Brazil, community health agents and physiotherapists interact with each other, and both play an important role in the health system. Community health agents can perform some of the functions of physiotherapists in the home, and working together the two groups can better meet the needs of patients with physical therapy needs by providing care in the home. |
| Pallas | 2013 | NSR | Scaling up and sustaining CHW programs in LMICs requires effective program design and management, including adequate training, supervision, motivation, and funding; acceptability of the program to the communities served; and securing support for the program from political leaders and health care providers. |
| Pereira | 2013 | NSR | In Brazil, community health agents require on-going health education in their daily work and in-service training on aspects of primary health care to enable them to consider all social determinants of health instead of taking a purely biomedical approach. The health care model must be reoriented to increase the communities’ involvement in their health. |
| Schneider | 2016 | SR | Between 2005 and 2014, there have been 678 publications on CHWs from 46 countries, with the annual publication rate increasing rapidly. Half the publications reported on initiatives in Africa, a third from Asia and 11% from the Americas (mostly Brazil). The largest single focus and driver of the growth in publications was on CHW roles in meeting the Millennium Development Goals of maternal, child and neonatal survival (35% of total), followed by HIV/AIDS (16%), reproductive health (6%), non-communicable diseases (4%) and mental health (4%). Only 17% of the publications approached CHW roles in an integrated fashion. There were also distinct regional (and sometimes country) profiles, reflecting different histories and program traditions. |
| Zulu | 2014 | NSR | Factors that may influence the integration of CHW programs into health systems in LMIC include: (1) incentives for CHWs that are consistent, predictable, appropriate and fair in relation to their tasks; (2) a workload that is considered reasonable; (3) good training, and (4) regular supervision from professional health workers. Different aspects of national CHW programs are integrated into the health system in various ways. The acceptability and adoption of national CHW programs by health systems has been shaped by the interaction between the perspectives of the actors within the adopting system as well as by the compatibility of CHWs with the health system. |

Three reviews examined the rights and well-being of LMIC CBPs (Table 8).

| Table 8. CBP rights and wellbeing-related LMIC reviews | | | |
| --- | --- | --- | --- |
| **1^st^ author** | **Date** | **Review type** | **Main outcomes and CBP roles** |
| Bhatia | 2014 | NSR | In India, the government has been slow to provide salary security to CHWs; CHWs are not integrated into the established, salaried team of health system workers. Performance-based incentives do not provide the financial security that is expected and needed by CHWs. |
| Henriques Camelo | 2012 | NSR | CHWs experience work-related physical and mental illnesses, such as circulatory, muscular and infectious diseases, mental disorders, stress, and burnout. The management strategies CHWs use include exercise, reading, music, and team meetings. Work-related conditions that CHWs experience must be reviewed, so that management strategies can be developed. CHWs need to reflect on their work-related practices and safety issues and how to minimize their health effects. |
| Kane | 2016 | NSR | CHWs programs empower CHWs by giving them (1) access to privileged medical knowledge, (2) linking them to the health system, and (3) providing them an opportunity to do meaningful and impactful work. However, CHWs are frustrated by (1) a sense of lack/absence of control over their work environment and (2) feelings of being unsupported, unappreciated, and undervalued. While increasingly the onus is on CHWs and CHW programs to solve the problem of health access, attention should be given to the experiences of CHWs themselves. CHW programs need to move beyond an instrumentalist approach to CHWs, and take a more inclusive approach that also includes the career developmental and empowerment of CHWs. |

Thirty-nine reviews addressed CBP involvement in maternal and child health initiatives (Table 9). Thirteen specifically focused on child and neonatal health, 15 on maternal and child health, five on vaccination, three on maternal health, and three on contraception. One of the reviews on maternal and child health (Glenton et al., 2013) was global in focus but included more LMIC than HIC literature so is presented below.

| Table 9. Maternal and child health LMIC reviews | | | |
| --- | --- | --- | --- |
| **1^st^ author** | **Date** | **Review type** | **Main outcomes and CBP roles** |
| **Child health** | | | |
| Amouzou | 2014 | SR | In sub-Saharan Africa, integrated community case management programs train lay CHWs to assess, classify and treat uncomplicated cases of pneumonia with antibiotics, malaria with antimalarial drugs, and diarrhea with oral rehydration salts and zinc. Six of the eight studies showed a higher decline in mortality among children aged two months to five years in program areas compared to comparison areas, although this acceleration was statistically significant in only one study with a decline of 76% larger in intervention than in comparison areas. |
| Bosch-Capblanch | 2014 | NSR | Integrated community case management programs, which aim to improve vulnerable communities' access to care (for childhood illnesses) through CBPs, seem to have positive effects when they involve: policy change, organizational change, standardization of clinical practices, alignment with other programs (especially large multi-component programs) and strong components of training, supervision, and supportive equipment and supplies. On-site training and supervision improve clinical practices. Positive effects are demonstrated on caregiver knowledge, care seeking behaviors, and household sickness management, but not on mortality. |
| Christo-pher | 2011 | SR | This review assessed the impact of CHWs delivering curative interventions against malaria, pneumonia and diarrhea on under-six mortality and morbidity in sub-Saharan Africa. The review found that one-to-four year-old mortality reduced by 63% when CHWs delivered insecticide-treated nets and 36% when CHWs delivered anti-malarial chemoprophylaxis in addition to curative treatment and education. However, there was limited information on program description, context, or process outcomes. Large-scale rigorous evaluations of CHW programs are needed. |
| de Oliveira Castro | 2015 | NSR | In Brazil, CHWs can play an important role in identifying children with hearing problems as well as promoting and monitoring children's hearing health. Effective training methods include live classes, online classes, video conferencing, and use of CD-ROMs. |
| Gogia | 2011 | SR MA | Community-based neonatal care provided by CHWs was associated with reduced neonatal mortality in resource-limited settings [RR=0.73 (0.65 to 0.83); P<0.0001]. In trials with a baseline neonatal mortality rate (NMR) less than 50/1000 live births, the relative risk of neonatal mortality among neonates receiving care from CHWs was 0.85 (0.73-0.99); while in trials with a baseline NMR of more than 50/1000 live births, the RR was 0.65 (0.54-0.77). Subgroup analysis by the type of intervention (i.e., home visits with or without community mobilization (RR 0.71 (0.60-0.84)), vs. community participatory action and learning (RR 0.77 (0.61-0.96)) indicate that both intervention strategies resulted in a similar reduced relative risk of neonatal mortality. While it appears logical that trials with more number of home visits should result in greater mortality reduction, this association was not consistently observed across all trials. Some studies suggest that home visits during the first 2 days of life are likely to yield the largest dividends. |
| Gogia | 2016 | SR MA | There is high quality evidence that home-based neonatal care is associated with a reduction in neonatal and perinatal mortality in South Asian settings with high neonatal mortality rates and poor access to health facility-based care. |
| Gogia | 2010 | SR MA | Home visits for antenatal and neonatal care, together with community mobilization activities, are associated with reduced neonatal mortality (RR: 0.62; 95% CI: 0.44–0.87) and stillbirths (RR: 0.76; 95% CI: 0.65–0.89) in south Asian settings with high neonatal mortality and poor access to facility-based health care. Antenatal and neonatal practice indicators also significantly improved (> 1 antenatal check-up, 2 doses of maternal tetanus toxoid, clean umbilical cord care, early breastfeeding and delayed bathing). Only one trial recorded infant deaths, and this study showed a marked reduction (RR: 0.41; 0.30–0.57). Subgroup analyses suggested a greater survival benefit when home visit coverage was ≥ 50% (P < 0.001) and when both preventive and curative interventions (including the use of injectable antibiotics) were conducted (P = 0.088). |
| Kane | 2010 | NSR | Interventions to improve CHW performance include: (1) skills-based training of CHWs, (2) a health system that provides rigorous CHW supervision and is responsive to the CHW’s client referrals, and (3) positioning the CHW in the community. When interventions were applied in context of CHW programs embedded in local health services, with beneficiaries who valued services and had unmet needs, the interventions worked if the following mechanisms were triggered: anticipation of being valued by the community; CHWs perceived that their social status improved; a sense of relatedness that CHWs had with beneficiaries and the health system; increase in self-esteem; sense of self-efficacy and mastery of tasks; an enhanced sense of credibility, legitimacy and assurance that there was a system for back-up support. Studies also showed that if context differed, even with similar interventions, negative mechanisms could be triggered, compromising CHW performance. |
| Lee | 2014 | SR MA | This review found that, compared to physicians, trained frontline health workers may screen for possible bacterial infection in young infants with relatively high sensitivity (average 82%; 95% CI 76%–88%) but somewhat lower specificity (average 69%; 95% CI 54%–83%) (eight studies, n= 11,857). Among the 14 studies that reported on health worker diagnosis of possible bacterial infection, five were about CBPs, and two of these five provided data that could be pooled for analysis. Both were from Bangladesh and validated CHW classification of newborns by modified Bangladeshi Integrated Management of Childhood Illness criteria compared to physician classification: 73%–91% of cases of very severe disease were recognized by CHWs, with specificity of 95%–98% (Baqui et al., 2009; Darmstadt et al., 2009). The third study, from Gadchiroli India, found that CHWs could identify individual signs of neonatal illness in high agreement with physicians (mean 92.7% agreement on 46 variables) and diagnosed 89% of cases meeting clinical sepsis criteria compared to a computer diagnostic algorithm based on neonatal symptoms (Bang et al., 2005; Bang, Bang, Baitule, Deshmukh, & Reddy, 2001). The fourth study, from Nepal, found that community-based CHWs had high levels of agreement on the major signs of neonatal sepsis compared to facility-based CHWs (Khanal et al., 2011). The fifth, from Purulia, India, was not as positive (Biswas, Mukhopadhyay, Mandal, Panja, & Sinha, 2011). This study observed CBPs conducting home assessments for Integrated Management of Neonatal and Childhood Illnesses. These CBPs completed all aspects of the assessment in only 32% of cases and, among those cases, 35% had the correct classification in all subgroups and 34% in at least one subgroup. |
| Noordam | 2015 | NSR | Among illiterate CHWs, the use of counting beads (beads that are shifted along a string to help keep track of numbers while counting) enabled and improved the assessment and classification of fast breathing among children with possible pneumonia. However, among literate CHWs, a study found that the use of counting beads decreased the accuracy of counting breaths. The design of the beads is crucial: beads should move comfortably, and a separate bead string, with color coding, is required for the age groups with different cut-off thresholds—eliminating more complicated calculations. |
| Reisman | 2016 | SR | Birth attendants in LMICs can acquire newborn resuscitation knowledge and skills through training, but they struggle to learn bag-mask ventilation, and there is a significant falloff in knowledge and skills after training. Refresher training, including formal, structured practice sessions, improves retention of knowledge and skills. Low rates of proficiency with bag-mask ventilation immediately after training suggest that educational programs should strengthen their emphasis on learning this technique, including methods of improving ventilation when it is initially ineffective. The review also highlights that birth attendants can acquire knowledge and skills simply by working with those who are trained rather than undergoing the training themselves, and that self-directed learning may be a viable and cost-effective strategy for improving newborn care in LMICs. |
| Sazawal | 2003 | SR MA | Community-based case management of pneumonia in children in developing countries involves diagnosis by CBPs through assessment of rapid breathing and treatment by CBPs with antibiotics. Meta-analysis found a reduction in neonatal mortality of 27% (95% CI 18–35%), infant mortality of 20% (11–28%), and children 0 to four years of 24% (14–33%). In the same three groups, pneumonia mortality was reduced by 42% (22–57%), 36% (20–48%), and 36% (20–49%), respectively. |
| Winch | 2005 | NSR | CHW pneumonia case management has the strongest evidence for an impact on mortality, compared to six other intervention models of care for children with malaria or pneumonia outside health facilities (e.g., CHW basic management and verbal referral; CHW basic management and facilitated referral; CHW directed fever management; family-directed fever management; CHW malaria management and surveillance; and CHW integrated multiple disease case management). In the CHW pneumonia case management model, CHWs assess child respiration, provide antibiotics for pneumonia, and referred children to health facilities. Pneumonia case management by CHWs is a child health intervention that warrants considerably more attention, particularly in Africa and South Asia. |
| **Maternal and child health** | | | |
| Darmstadt | 2009 | SR MA | There is evidence of moderate quality that CHWs have a positive impact on perinatal/neonatal outcomes. CHWs can play a promising role providing pregnancy and childbirth care, mobilizing communities, and improving perinatal outcomes. There is low-to-moderate evidence that training TBAs improves linkages with health facilities and perinatal outcomes. The evidence for providing skilled birth care in the community is of low quality, but shows a 12% reduction in perinatal mortality and a 22-47% reduction in intra-partum related neonatal mortality. |
| Dawson, Brodie, et al. | 2014 | NSR | Between-country (HIC-LMIC) collaborations are useful for strengthening midwifery capacity (clinical-and-research-skill building, the development of tailored education programs and the establishment of structures and systems to enhance the midwifery workforce). Collaborations are strengthened through the establishment of clear processes for communication, leadership and appropriate membership, effective management, mutual respect, as well as an understanding of the context. |
| Dawson, Buchan, et al. | 2014 | SR | Lay health workers were able to provide injectable contraception effectively - with high quality and with high levels of patient satisfaction. Collaborative approaches involving community members and health workers at all levels have the potential to deliver maternal and reproductive health interventions effectively if accompanied by ongoing investment in the health care system. |
| Gilmore | 2013 | SR | There is some evidence of moderate quality that CHWs are effective in malaria prevention, health education, breastfeeding promotion, promotion of essential newborn care, and psychosocial support. CHWs are particularly effective for promoting mother-performed strategies such as skin-to-skin care for newborns and exclusive breastfeeding. The evidence is insufficient to draw conclusions for most interventions. More research is needed. |
| Giugliani | 2011 | SR | Community health agents (CHAs) in Brazil have demonstrated effectiveness in increasing the frequency of child weighings, the prevalence of breastfeeding, and delaying the introduction of bottle feeding. There is lower-quality evidence the CHAs are having an effect on infectious and non-communicable diseases as well as on reducing inequities. Given the extent of CHA utilization in Brazil (being one of the largest CHW programs in the world and reaching a major segment of the population), more research is needed on CHA effectiveness. |
| Glenton, Colvin^[[5]](#footnote-5)^ | 2013 | SR MA | Lay health workers (LHWs) in HICs mainly offered promotion, counseling and support while in LMICs they offered similar services but sometimes also distributed supplements, contraceptives and other products, diagnosed and treated children with common childhood diseases, and managed uncomplicated labor/referrals. Program recipients appreciate LWHs’ skills and the similarities between themselves and the LHWs; health professionals appreciated LHWs’ commitment, their contributions in reducing workload and their communication skills. However, some recipients were concerned about confidentiality or saw LHW services as not relevant or not sufficient, and some providers felt LHWs increased their workload or could cause providers to lose authority – they also worried that LHWs trained to manage uncomplicated delivery were overconfident. LHWs and recipients emphasized the importance of trust, respect, kindness and empathy. However, LHWs sometimes found it difficult to manage emotional relationships with recipients, feared blame, or were demotivated when their services were not appreciated. Those trained to manage labor sometimes faced patient resistance and health system shortcomings when referring women to facilities. LHWs required higher quality training, improved supervision and opportunities to share experiences with other LHWs or voice complaints. LHWs required and valued support from local leaders, the health system, and their families and were motivated by factors including altruism, social recognition, knowledge gain and career development. The authors found a range of opinions on remuneration, including that some unsalaried LHWs wanted regular payment, while others were concerned that payment might threaten their social status and that some salaried LHWs were dissatisfied with their pay levels or frustrated by inconsistent payment. |
| Lassi^#^ | 2015 | SR MA | The meta-analysis found that community-based intervention packages have a robust effect on reducing neonatal deaths (pooled analysis demonstrates a 25% reduction in overall neonatal deaths, 19% reduction in stillbirths and 22% reduction in perinatal mortality) and may have a possible effect on reducing maternal mortality, although the pooled result just crossed the line of no effect. Packages that disseminated education and promoted awareness related to birth and newborn care preparedness through community/women’s groups were best for reducing deaths throughout the neonatal period as well as during the early neonatal period. On the other hand, packages that comprised community mobilization and education strategies and home visitation by CHWs managed to reduce neonatal, perinatal deaths and stillbirths, possibly because these strategies focused on women in the antenatal period and on early newborn care as well as on the management and referral of sick newborns. When community mobilization was added to home-based neonatal care by CHWs, it significantly reduced total neonatal deaths by 44% (according to one study). When traditional birth attendants (TBAs) visited homes with formally trained midwives, stillbirths declined by 46%, whereas when TBAs visited homes alone no reduction in stillbirths was observed. |
| Lewin | 2010 | SR MA | The use of lay health workers, compared to usual healthcare services: (1) probably increases breastfeeding and up-to-date childhood immunization (meta-analysis for immunization: RR 1.23, 95% CI 1.09 to 1.38; P = 0.0006, but heterogeneous effects I2 = 70%, P = 0.005), (2) may increase the number of parents who seek help for their sick child, and (3) may lead to slightly fewer children who suffer from fever, diarrhea and pneumonia as well as to (4) fewer deaths among children younger than five years of age. The use of lay health workers, compared to people helping themselves or going to a clinic, probably leads to an increase in the number of people with tuberculosis who are cured and probably makes little or no difference in the number of people who complete preventive treatment for tuberculosis. |
| Miyake | 2017 | NSR | In fragile and conflict-affected states, community linkages and acceptance are enablers of community-level midwifery services that produce improve skilled care. Barriers to improving such care include inappropriate recruitment, non-standardized education, a weak supportive environment, as well as political insecurity and violence. |
| Prost | 2014 | SR MA | Women’s groups practicing participatory learning and action, compared with usual care, have a positive impact on birth outcomes in low-resource settings. Meta-analyses of seven trials showed that exposure to women’s groups was associated with a 37% reduction in maternal mortality (odds ratio 0.63, 95% CI 0.32–0.94), a 23% reduction in neonatal mortality (0.77, 0.65–0.90), and a 9% non-significant reduction in stillbirths (0.91, 0.79–1.03). Meta-regression found that increased proportion of pregnant women in groups was linearly associated with reduction in both maternal and neonatal mortality. Analysis of the four studies in which at least 30% of pregnant women participated in groups showed a 55% reduction in maternal mortality (0.45, 0.17–0.73) and a 33% reduction in neonatal mortality (0.67, 0.59–0.74). The intervention was cost-effective by WHO standards and could save an estimated 283 000 newborn infants and 41 100 mothers per year if implemented in rural areas of 74 priority LMIC countries. |
| Ribeiro Sarmento | 2014 | NSR | Trained TBAs have performed a wide variety of tasks, including outreach and case finding, health and patient education, referrals, home visits and care management. TBA training was significantly associated with higher numbers of referrals and greater use of facility-based care by women with obstetric complications. TBAs can identify early signs of complications during labor and delivery, and they can successfully refer mothers for treatment in health centers by skilled health workers. |
| Sibley | 2006 | SR MA | In settings characterized by high mortality and weak health systems, trained TBAs can contribute to reducing mortality through participation in key evidence-based interventions. Compared to untrained TBAs, pooled meta-analysis showed that trained TBAs had a 44% increase over baseline in safe delivery (effect size: 0.35; 95% CI: 0.19-0.51), 103% increase in clean delivery (effect size: 0.72; 95% CI: 0.49-0.96), 53% increase in clean cord-care practices (effect size: 0.41; 95% CI: 0.24-0.57); 117% increase in knowledge of appropriate antenatal referral (effect size 0.97; 95% CI: 0.40-1.55); 47% increase in appropriate referral for antenatal issues (effect size 0.39; 95% CI 0.12-0.67); 36% increase in appropriate referral for obstetric complications (effect size 0.39; 95% CI 0.15-0.45). Trained TBAs showed significantly improved counseling behavior on maternal nutrition, early exclusive breastfeeding, and immunization (primarily tetanus toxoid immunization) (effect size 15%). TBA training is associated with small but significant decreases in perinatal mortality and neonatal mortality due to birth asphyxia and pneumonia. TBAs have been trained to act as a link to more formally trained skilled birth attendants. Some have been trained to upgrade their skills and perform safe deliveries, and others have taken on expanded functions including preventive services, screening and referral. |
| Sibley | 2012 | SR MA | There remains insufficient evidence to establish the potential of TBA training to improve perinatal and neonatal mortality. However there is evidence from one cluster randomized controlled trial that trained TBAs reduce perinatal mortality, stillbirths, and neonatal mortality. In a meta-analysis comparing TBAs who received additional training in resuscitation of newborns with TBAs who had received only basic training there was no significant difference in stillbirths or in early neonatal mortality. The results are promising for some outcomes (perinatal mortality, stillbirths and neonatal mortality) but more studies are needed. Trained TBAs can provide advice on use of antenatal iron and folic acid tablets or they can actually distribute them. They can provide advice on or distribute anti-malarials. They can advise on or distribute vitamin A. They can provide advice on tetanus vaccination, on early initiation of breastfeeding and exclusive breastfeeding during the first 6 months of life, on referral to a health facility, and on the use of antenatal and postnatal care as well as the use of family planning. |
| Silveira Feyer | 2013 | NSR | In Brazil, TBAs have entered into their careers on the basis of family tradition, feelings of solidarity, divine calling, or simply as a response to community needs. Virtues of a good TBA include patience, courage, respect, generosity of spirit, and perseverance. The TBAs’ knowledge and practices involved, among others, the use of herbal teas and maneuvers at different stages of delivery. Difficulties faced by some TBAs include being far from professional support, difficult working conditions, and lack of pay. The Brazilian health system has provisions now for paying those who attend deliveries, but TBAs struggle to access this remuneration. |
| Wilson | 2012 | SR MA | Incorporating training on maternal, perinatal and neonatal health as well as support of TBAs in developing countries reduces perinatal mortality (relative risk 0.76, 95% confidence interval 0.64 to 0.88, P<0.001) and neonatal death (relative risk 0.79, 95% confidence interval 0.69 to 0.88, P<0.001. Meta-analysis of the non-randomized studies also showed a significant reduction in perinatal mortality (relative risk 0.70, 95% confidence interval 0.57 to 0.84, p<0.001) and neonatal mortality (relative risk 0.61, 95% confidence interval 0.48 to 0.75, P<0.001). |
| **Vaccination** | | | |
| Corluka | 2009 | SR | This review sought to determine whether vaccination programs delivered by CHWs are cost-effective. Although the studies were methodologically strong, none adequately addressed affordability and sustainability, so no conclusions on cost-effectiveness could be drawn. |
| Glenton^[[6]](#footnote-6)^ | 2011 | SR MA | Most of the studies showed that CHWs increased immunization coverage. A meta-analysis of four studies showed that CHW promotion of vaccination increased the proportion of children with up-to-date vaccinations (relative risk: 1.19, 95% CI: 1.09-1.30). For the two studies where LHWs actually provided immunization (in Papua New Guinea and Guatemala), both showed an improvement compared to a control group, but the evidence was of low quality. The remaining six studies, in which CHWs promoted immunization, showed no consistent findings. |
| Glenton, Khanna | 2013 | SR | There is evidence, but of low-quality, that health professionals are confident that CHWs can deliver vaccines or other medicines using compact pre-filled auto-disposal devices (CPADs) to mothers and children. Some health professionals said providing adequate supervision was difficult. CHWs perceived CPADs as effective and important, but feared consequences if harm should come to the recipients. No studies have yet assessed side effects or safety. |
| Oyo-Ita | 2016 | SR MA | There is evidence of moderate certainty that health education at village meetings or at homes (in some cases provided by CBPs) probably improves coverage with three doses of diphtheria-tetanus-pertussis vaccines (DTP3: risk ratio (RR) 1.68, 95% confidence interval (CI) 1.09 to 2.59). Regular immunization outreach (by nurse plus assistant – unclear if this includes a CBP) may improve full immunization coverage (RR 3.09, 95% CI 1.69 to 5.67, low-certainty evidence), which may substantially improve if combined with conditional cash transfer incentives to households (RR 6.66, 95% CI 3.93 to 11.28, low-certainty evidence). Home visits to identify non-vaccinated children and refer them to health clinics may improve uptake of three doses of oral polio vaccine (RR 1.22, 95% CI 1.07 to 1.39, low-certainty evidence). There was low-certainty evidence that integration of immunization with other services may improve DTP3 coverage (RR 1.92, 95% CI 1.42 to 2.59). |
| Patel | 2010 | NSR | While the limited number and poor quality of available studies make it difficult to directly compare CHW interventions to other strategies for improving immunization coverage, it is clear that CHWs make diverse contributions toward strengthening immunization programs. The roles CHWs played in interventions to increase vaccination included: compiling lists or making maps of children requiring vaccination, motivating parents, and following up with vaccine defaulters. Interventions involving CHWs had a mean increase in coverage of 20%. CHWs are well-suited to aid in identifying, tracking, and providing outreach services (especially to marginalized groups) as well as in providing information, education, and other communications to community members. |
| **Maternal health** | | | |
| Byrne | 2011 | SR | Integration of traditional birth attendants (TBAs) with the formal health system can increase skilled birth attendance. Mechanisms for integration include training and supervising TBAs, collaboration skills for health workers, inclusion of TBAs at health facilities, communication systems, and clear definition of roles. Success in increasing skilled birth attendance was context-dependent (e.g., the process for selection of TBAs, level of community participation, and reducing barriers to accessing the health system). |
| Rahman | 2013 | SR MA | In LMICs, the burden of common perinatal mental disorders can be reduced through mental health interventions delivered by supervised non-specialists and community workers (pooled effect size of -0.38, 95% CI: -0.56 to -0.21; F=79%). Local, trained CHWs were effective in delivering psychosocial and educational interventions to reduce maternal depression. |
| Smith | 2016 | NSR | All three large trials of community distribution of misoprostol included in this review found that administration of misoprostol by Lady Health Workers (LWS) to women in their care was associated with some reduction in bleeding or in serum hemoglobin (a measure of anemia) post-partum compared with placebo or standard care. Eleven trials implemented advance distribution of misoprostol, wherein pregnant women received misoprostol from LHWs during their pregnancy and were counseled (along with family members) on its use. These studies show that advance distribution of misoprostol for post-partum hemorrhage appears manageable with minimum risk and that the benefits of self-administration, especially for women who have little chance of expert care for post-partum hemorrhage, are considerable. Key programmatic considerations include the following: (1) LHWs should receive adequate training on misoprostol and how to deliver information on its use to women, (2) illiterate LHWs need to have access to sufficiently intensive training, (3) an adequate supply of misoprostol should be available, (4) appropriate monitoring and reporting mechanisms should be in place for drug supply and potential misuse, LHW capacity and motivation to reach remote community members, community acceptance of misoprostol, and the need to ensure that misoprostol does not influence women’s uptake of facility delivery. |
| **Contraception** | | | |
| Bellows | 2015 | NSR | There is some evidence that performance-based incentives for CBPs (primarily based on sales commission or payment for referral) can increase family planning uptake at the community level, but results are mixed and more research is needed. Careful attention must be paid to ethical issues and to ensuring non-coercion. |
| Malarcher | 2011 | SR | The injectable contraceptive depot-medroxyprogesterone acetate (DMPA) can be provided safely in communities by appropriately trained and supervised CHWs. The benefits outweigh the potential harms. Trained CHWs had sufficient knowledge and skills for screening potential clients regarding their eligibility for the use of DMPA. Clients of CHWs receiving DMPA had outcomes equivalent to those of clients of clinic-based providers of progestin-only injectables and were satisfied with community-based provision of DMPA. |
| Scott | 2015 | SR | Of 56 studies, 93% indicated that CHW family planning programs effectively increased the use of modern contraception, while 83% reported an improvement in knowledge and attitudes concerning contraceptives. CHWs were able to provide counseling on contraceptives, provide contraceptives, and refer to health facilities for more specialized care. |

Five reviews focused on CBP involvement in non-communicable diseases in LMICs: one on cancer and four on mental health (Table 10).

| Table 10. Non-communicable disease LMIC reviews | | | |
| --- | --- | --- | --- |
| **1^st^ author** | **Date** | **Review type** | **Main outcomes and CBP roles** |
| **Cancer** | | | |
| Wadler | 2011 | NSR | CHWs could assume three main roles along the cancer control continuum: health education, screening, and patient navigation. By raising awareness about breast cancer through education, women are more likely to undergo screening. Many more women can be screened, resulting in earlier-stage disease if CHWs are trained to perform clinical breast exams. |
| **Mental health** | | | |
| Mutamba | 2013 | SR MA | Three adult study populations provided evidence that CHW-led interventions are effective for reducing the burden of mental, neurological and substance-use disorders, including depression and post-traumatic stress disorder. Four of the studies targeting child mental health outcomes – one using selective prevention (targeting a higher-risk subgroup) and three using indicated prevention (targeting individuals identified as at-risk) –showed that the interventions were effective. CHW roles included providing psychosocial stimulation to children (in some cases along with nutritional supplements), emotional and social support, psychotherapy and counseling, as well as improving education and awareness. |
| Stacciarini | 2012 | NSR | *Promotoras* (CHWs) empower community members to promote mental health and prevent exacerbations of mental illness. *Promotoras*, when trained carefully, have the ability to increase awareness and to promote mental health in populations that would otherwise have limited or no access to care. *Promotoras* provide education, act as mediators between individuals and the health care system, advocate for both individual and community needs, and serve as role models for positive behaviors. |
| Singla | 2017 | SR MA | Non-specialist providers, usually CHWs or peers, are more effective than usual care or delayed treatment (waitlisted) groups in the provision of mental health treatments. Most treatments targeted depression or post-traumatic stress. Treatments were usually delivered with fewer than 10 sessions over two to three months in an individual, face-to-face format in community or primary-care settings. Treatments included common elements, such as nonspecific engagement and specific domains of behavioral, interpersonal, emotional, and cognitive elements. The pooled effect size was 0.49 (95% confidence interval = 0.36–0.62), favoring interventions provided by non-specialist providers (including CHWs). The provision of mental health care by non-specialist providers is more effective than usual care and improves access to mental health care. |
| van Ginneken | 2013 | SR MA | Non-specialist health workers (NSHWs), a classification that includes both professionals (e.g., doctors, nurses, and social workers) and lay health workers working at the primary care or community level, have some promising benefits in improving people’s outcomes for general and perinatal depression, post-traumatic stress disorder and alcohol-use disorders, and patient- and carer-outcomes for dementia. Services provided by NSHWs, compared with usual healthcare services, may increase the number of adults who recover from depression or anxiety (or both) two to six months after treatment; may slightly reduce symptoms for mothers with depression; may slightly reduce the symptoms of adults with post-traumatic stress disorder; probably slightly improve the symptoms of people with dementia; probably improve or slightly improve the mental well-being, burden and distress of carers of people with dementia; and may decrease the quantity of alcohol consumed by problem drinkers. This evidence is mostly of low or very low quality, and for some issues no evidence is available. In most studies NSHWs delivered the mental health care and addressed depression or anxiety (or both) or post-traumatic stress disorder. NSHWs can provide follow up to check on adherence, effects of medications, and side effects. Of the 38 studies in the review, 22 used lay health workers. |

Sixteen reviews focused on CBP involvement in infectious disease in LMICs: seven on HIV, six in malaria, one each on Buruli Ulcer, TB and neglected tropical diseases. In addition, four reviews on HIV (Flynn et al., 2017; Hall et al., 2017; Ma et al., 2016; Tso et al., 2016) had a global focus but included primarily LMIC literature and are thus also presented below (Table 11).

| Table 11. Infectious disease LMIC reviews | | | |
| --- | --- | --- | --- |
| **1^st^ author** | **Date** | **Review type** | **Main outcomes and CBP roles** |
| **HIV** | | | |
| Bemelmans | 2016 | NSR | In sub-Saharan Africa, lay counselors play a critical role in scaling up HIV services and addressing gaps in the HIV testing and treatment cascade by providing HIV testing and counseling and adherence support at both the facility and community levels. Countries have taken various steps in recognizing lay counselors, including (in Lesotho, Mozambique and Zimbabwe) well-defined harmonizing training, job descriptions, support structures for supervision and remuneration and (in Lesotho, Mozambique, Zambia and Zimbabwe) Ministry of Health certification for HIV testing services training. However, formal integration of this cadre into national health systems is limited, as lay counsellors are usually not included in national health workforce strategies or budgeting. |
| Campbell | 2011 | NSR | "Community embeddedness" has been overlooked in the WHO report on task shifting for HIV. Community embeddedness is required for CHWs to successfully perform socially embedded tasks such as health education and counseling, which are critical for HIV programming. The literature review identified six lessons for CHW success: (1) need for strong management, (2) appropriate selection of CHWs (3) suitable training, (4) adequate retention structures, and (5) good relationships with other healthcare workers, and (6) "community embeddedness." |
| Flynn^[[7]](#footnote-7)^ | 2017 | SR | Of the 50 countries analyzed, 58% either do not permit lay providers to perform HIV rapid diagnostic tests using fingerstick blood (the most common type) or do not specify if they can, while 44% do not permit or do not specify whether lay providers can perform HIV pre- and post-test counseling. In addition, fewer than half (46%) of the reports from countries to the Global AIDS Response Progress Reporting provided data that were consistent with their corresponding national HIV testing policy. Greater care must be taken when reporting Global AIDS Response Progress Reporting data to make sure it correlates with approved practice. Africa has a more supportive policy environment for lay provider HIV testing services compared to other global regions. Given the low uptake of lay provider use globally and their proven use in increasing HIV testing, countries should consider revising policies to support lay provider testing using rapid diagnostic tests. |
| Hall^7^ | 2017 | SR MA | Task shifting to lay health workers was generally acceptable to individuals living with HIV. Lay counselors were able to spend more time with individuals living with HIV, provided more social and non-medical instrumental support (such as arrangement of rides to clinic). Lay health worker counseling and peer health education nurtured hope and a positive attitude, which increased retention in care. |
| Kredo | 2014 | SR MA | Shifting responsibility from doctors to adequately trained and supported CHWs for managing HIV patients probably does not decrease the quality of care. |
| Ma^7^ | 2016 | SR MA | Task shifting was acceptable to persons living with HIV infection (PLHIV), and lay health workers can resolve the shortage of medical professionals for HIV care, strengthen the relationship between the community and the health system, improve the psychosocial wellbeing of PLHIV, and empower them to achieve better adherence. Proper training and compensation for lay health workers can better facilitate task shifting. |
| Mdege | 2013 | SR | Task shifting from health care professionals to lay health worker for antiretroviral treatment achieved equivalent patient outcomes in terms of mortality, viral load, CD4 cell count, adherence to treatment, loss to follow-up, health care utilization, occurrence of new AIDS-defining illnesses, incidence of opportunistic infections, toxicity of antiretroviral treatment, quality of life, and other measures of treatment failure. However, most of the identified studies in the review were underpowered. Task-shifting resulted in substantial cost and physician time savings. |
| Mwai | 2013 | SR | CHWs were reported to enhance the reach, uptake and quality of HIV services, as well as the dignity, quality of life and retention in care for people living with HIV. The presence of CHWs in clinics was reported to reduce waiting times, streamline patient flow and reduce the workload of health workers. Clinical outcomes appeared not to be compromised, with no differences in virologic failure and mortality when comparing patients receiving community-based care with receiving facility-based care. |
| Petersen | 2014 | SR | South African lay counselors working in chronic care have poorly defined roles, inconsistent remuneration, no standardized training, as well as poor supervision and logistical support. Studies provide evidence that under controlled conditions with adequate training and supervision, lay-counselor behavior change counseling interventions using various adaptions of the information-motivation-behavioral skills model can reduce HIV-risk behaviors including unprotected sex, alcohol use before sex, number of sexual partners; and transactional sex. |
| Tso^7^ | 2016 | SR MA | Effective interventions that link people living with HIV to care include: task shifting (4 studies, high confidence rated using CERQual, a tool analogous to GRADE for evaluating the confidence of qualitative systematic review findings), community-based mobile outreach testing and linkage (3 studies, high confidence), integration of HIV-specific and primary medical care (2 studies, moderate confidence), provider initiated testing, counseling and linkage (2 studies, low confidence), and cessation support for people who use illicit drugs to prepare for HIV treatment [??] (2 studies, high confidence). Lay health workers generally found these interventions acceptable but were hindered by insufficient administrative support and inadequate training. |
| Wouters | 2012 | SR | In task-shifting to support antiretroviral treatment (ART), CHWs can provide psycho-social care, help trace treatment defaulters, serve as peer counselors and adherence supporters to increase compliance with ART. Task-shifting to CHWS had a definite positive impact on a wide range of program quality indicators, including access, coverage, adherence, virological and immunological outcomes, patient retention, and patient survival. |
| **Malaria** | | | |
| Boyce | 2017 | SR | Rapid diagnostic tests are performed safely by CBPs when proper training is provided. Rapid diagnostic tests are executed by CHWs with high sensitivity and specificity, and CHWs display high levels of adherence to treatment guidelines. Several of the included studies showed that supplying CHWs with additional training and job aids significantly improved their performance of rapid diagnostic test procedures. |
| Kabaghe | 2016 | SR MA | In the three studies that assessed CBP performance in malaria diagnosis and management, CBPs provided appropriate treatment for 98% of the cases. Those testing positive were treated 99% of the time and those testing negative were not treated 94% of the time. Improving health worker compliance to negative malaria rapid diagnostic test results will prevent the mismanagement of patients and overprescribing of anti-malarial drugs. Improving diagnostic capacity for other febrile illnesses and developing local evidence-based guidelines may help improve compliance and management of negative rapid diagnostic test results. |
| Kamal-Yanni | 2012 | NSR | CHWs can effectively diagnose and treat malaria and other common fevers, even in remote areas. CHWs correctly used rapid diagnostic tests, dispensed artemisinin-based combination therapies (with substantially lower rates of over-prescription compared to healthcare providers in public and private facilities), counseled patients, and performed effective case management of child pneumonia. CHWs scored well in their knowledge and practices of treatment provision in most studies. CHWs increased treatment coverage in underserved areas and reduced delays in care seeking. Their role should be recognized and expanded. The evidence shows that there is no short-cut to investing in training and supervision of providers and that malaria treatment must be integrated into the public health system (rather than treated through vertical programs). |
| Paintain | 2014 | SR | CHWs are able to provide good-quality malaria care, including performing procedures such as rapid diagnostic tests (RDTs). Appropriate training, clear guidelines, and regular supportive supervision are important facilitating factors. Crucial to sustainable success of CHW programs is strengthening health system capacity to support the supply of commodities used by CHWs, supervision of CHWs, and appropriate treatment of cases referred by CHWs. Study findings support the notion that pre-packaged antimalarial drugs can be administered effectively by CHWs and adhered to by those receiving these medications. Appropriate treatment at the community level according to RDT result was higher than that found in a number of studies of RDT use by more highly trained health workers located at facilities, who inappropriately treated 30-80% of RDT-negative patients with antimalarial drugs. In contrast to the positive findings on quality of malaria case management, the evidence from this review suggests a more mixed performance for community-level management of pneumonia by CHWs in the context of integration with malaria diagnosis and treatment. |
| Ruizendaal | 2014 | SR | For community case management of malaria, CHWs were able to correctly perform rapid diagnostic tests (RDTs), although specificity levels were variable. CHWs showed high adherence to test results, but in some studies a substantial group of patients who were RDT-negative received treatment. Because of the poor quality of the studies, no effect on morbidity or mortality could be estimated. Uptake and acceptance by the community was high. However, patients with negative RDT results did not always follow up referral advice. Drug or RDT stock-outs and limited information on CHW motivation are bottlenecks for sustainable implementation. |
| Sunguya | 2017 | SR | CBPs had important preventive, case management, and promotive roles in malaria interventions, including health surveillance and health promotion specific to malaria. However, CBPs faced many challenges in implementing integrated community case management for malaria. These challenges included (1) poor and unsustainable financing for integrated community case management for malaria, (2) workforce-related issues (including short-term training, which reduced community confidence in CBPs), (3) lack of and unstable supply of medicines and diagnostics, (4) lack of information and research, and (5) challenges related to service delivery and leadership. |
| **Buruli ulcer** | | | |
| Vouking Zambu | 2013 | NSR | CHW programs can have large impacts on the control of Buruli ulcer in sub-Saharan Africa. However, larger numbers of CHWs are needed in order to improve the detection and management of cases. One of the major obstacles to the control of Buruli ulcer is inadequately staffed and poorly-equipped health facilities in the affected areas. |
| **TB** | | | |
| Wu | 2017 | NSR | Three of the 21 included studies on TB healthcare training programs contained information on lay health workers: one was experiential hands-on training and two were lecture-based. Data on the effectiveness of these training programs was not presented but the authors conclude that although significant funds have been invested in TB health care provider training, publications of robust evaluations assessing the impact on quality of care and behaviour change are limited. |
| **Neglected tropical diseases** | | | |
| Corley |  | SR | CHWs and nurses have contributed to the control and near eradication of a number of neglected tropical diseases in sub-Saharan Africa. CHWs are important for enabling meaningful engagement and participation of communities in neglected tropical diseases programs that are successful and sustainable. Expanded roles for neglected tropical diseases are needed in the future. Key lessons are: (1) successful disease control requires deep and meaningful engagement with local communities; (2) expanding the role of nurses and CHWs will be required if sub-Saharan African countries are to meet neglected tropical disease treatment goals and eliminate the possibility future disease transmission; and (3) horizontal disease control programs can create complementary interactions between their different control activities as well as reduce costs through improved program efficiencies—benefits that vertical programs are not able to attain. There exists a growing body of literature to suggest that interventions against conditions such as malaria, diarrheal diseases, malnutrition, and a whole host of neglected tropical diseases can realistically be managed at the community level by well-trained and well-managed CHWs. Future neglected tropical diseases control programs should be thoughtfully crafted, well-integrated horizontal programs that operate parallel to and in harmony with other primary- care-health efforts. |

No studies on CBP involvement in palliative community-based care were found that met Horey et al.’s (2015) meta-analysis inclusion criteria. They concluded that there is an absence of evidence to show how best to train or support palliative care volunteers whilst maintaining standards of care for palliative care patients and their families.

One study was classified as “LMIC other” and focused on adolescent health (Table 12).

Table 12. Other LMIC reviews

| **1^st^ author** | **Date** | **Review type** | **Main outcomes and CBP roles** |
| --- | --- | --- | --- |
| Koon | 2013 | NSR | This review examined the potential of CHWs for strengthening the delivery of adolescent health services in sub-Saharan Africa. A single study used CHWs to deliver adolescent health services with promising results. CHW programs involving the delivery of adolescent health services have proliferated despite the absence of high quality evaluations. Further research is needed, including on the capacity and comparative effectiveness of generalist versus specialist CHW programs. |

1. This article had a global scope but the majority of the included studies were from HIC settings [↑](#footnote-ref-1)
2. This article had a global scope but the included studies appear to be from HIC settings [↑](#footnote-ref-2)
3. This article had a global scope but the majority of the included studies were from HIC settings [↑](#footnote-ref-3)
4. No regional focus mentioned but the included article appears to be from HICs [↑](#footnote-ref-4)
5. Seventeen of the LHW programs were based in low income countries (Bangladesh, Ethiopia, Gambia, Kenya, Malawi, Nepal, Uganda, Viet Nam, Zambia, Zimbabwe); 19 were based in middle income countries (Brazil, Ghana, Guatemala, Honduras, India, Iran, Mexico, Nicaragua, Pakistan, Papua New Guinea, South Africa, Thailand); and 17 were based in HICs (Australia, Canada, USA, UK). [↑](#footnote-ref-5)
6. Glenton et al. (2011) included seven studies from HICs and five from LMICs. We have included it in the LMIC section to cluster the studies on CBP involvement in vaccination coverage.

   # Lassi et al. included 26 studies, of which one was from a HIC (Greece) [↑](#footnote-ref-6)
7. These review articles had a global scope but the majority of the included studies were from LMIC settings [↑](#footnote-ref-7)
