# Supplementary material for: What do we know about community-based health worker programs? A systematic review of existing reviews on community health workers
Source: Hum Resour Health. 2018 Aug 16;16:39. doi: 10.1186/s12960-018-0304-x (PMC6097220; doi:10.1186/s12960-018-0304-x)
Supplement: Supplementary file 5 — Included and excluded articles. (DOCX 55 kb) [file 12960_2018_304_MOESM5_ESM.docx]

**Appendix 5. Included and excluded articles**

**Included articles (n=122)**

Abbott, L. S., & Elliott, L. T. (2017). Eliminating Health Disparities through Action on the Social Determinants of Health: A Systematic Review of Home Visiting in the United States, 2005–2015. *Public Health Nursing*, *34*(1), 2–30. https://doi.org/10.1111/phn.12268

Agarwal, S., Perry, H. B., Long, L. A., & Labrique, A. B. (2015). Evidence on feasibility and effective use of mHealth strategies by frontline health workers in developing countries: Systematic review. *Tropical Medicine and International Health*, *20*(8), 1003–1014. https://doi.org/10.1111/tmi.12525

Amouzou, A., Morris, S., Moulton, L. H., & Mukanga, D. (2014). Assessing the impact of integrated community case management (iCCM) programs on child mortality: Review of early results and lessons learned in sub-Saharan Africa. *Journal of Global Health*, *4*(2), 20411. https://doi.org/10.7189/jogh.04.020411

Baatiema, L., Sumah, A. M., Tang, P. N., & Ganle, J. K. (2016). Community health workers in Ghana: the need for greater policy attention. *BMJ Global Health*, *1*(4), e000141. https://doi.org/10.1136/bmjgh-2016-000141

Bellows, N. M., Askew, I., & Bellows, B. (2015). Review of performance-based incentives in community-based family planning programmes. *The Journal of Family Planning and Reproductive Health Care / Faculty of Family Planning & Reproductive Health Care, Royal College of Obstetricians & Gynaecologists*, *41*(2), 146–51. https://doi.org/10.1136/jfprhc-2014-100883

Bemelmans, M., Baert, S., Negussie, E., Bygrave, H., Biot, M., Jamet, C., … Ford, N. (2016). Sustaining the future of HIV counselling to reach 90-90-90: A regional country analysis. *Journal of the International AIDS Society*. https://doi.org/10.7448/IAS.19.1.20751

Bhatia, K. (2014). Community health worker programs in India: a rights-based review. *Perspectives in Public Health*, *134*(5), 276–282. https://doi.org/10.1177/1757913914543446

Bornstein, V. J., & Stotz, E. N. (2008). Concepções que integram a formação e o processo de trabalho dos agentes comunitários de saúde: uma revisão da literatura. *Ciência & Saúde Coletiva*, *13*(1), 259–268. https://doi.org/10.1590/S1413-81232008000100029

Bosch-Capblanch, X., & Garner, P. (2008). Primary health care supervision in developing countries. *Tropical Medicine and International Health*, *13*(3), 369–383. https://doi.org/10.1111/j.1365-3156.2008.02012.x

Bosch-Capblanch, X., Liaqat, S., & Garner, P. (2011). Managerial supervision to improve primary health care in low- and middle-income countries (Review). *Cochrane Database of Systematic Reviews*, (9). https://doi.org/10.1002/14651858.CD006413.pub2.www.cochranelibrary.com

Bosch-Capblanch, X., & Marceau, C. (2014). Training, supervision and quality of care in selected integrated community case management (iCCM) programmes: A scoping review of programmatic evidence. *Journal of Global Health*, *4*(2), 20403. https://doi.org/10.7189/jogh.04.020403

Boyce, M. R., & O’Meara, W. P. (2017). Use of malaria RDTs in various health contexts across sub-Saharan Africa: a systematic review. *BMC Public Health*, *17*(1), 470. https://doi.org/10.1186/s12889-017-4398-1

Braun, R., Catalani, C., Wimbush, J., & Israelski, D. (2013). Community Health Workers and Mobile Technology: A Systematic Review of the Literature. *PLoS ONE*, *8*(6), 4–9. https://doi.org/10.1371/journal.pone.0065772

Brownstein, J. N., Chowdhury, F. M., Norris, S. L., Horsley, T., Jack, L., Zhang, X., & Satterfield, D. (2007). Effectiveness of Community Health Workers in the Care of People with Hypertension. *American Journal of Preventive Medicine*, *32*(5), 435–447.

Byrne, A., & Morgan, A. (2011). How the integration of traditional birth attendants with formal health systems can increase skilled birth attendance. *International Journal of Gynecology and Obstetrics*, *115*(2), 127–134. https://doi.org/10.1016/j.ijgo.2011.06.019

Campbell, C., & Scott, K. (2011). Retreat from Alma Ata? The WHO’s report on Task Shifting to community health workers for AIDS care in poor countries. *Global Public Health*, *6*(2), 125–138. https://doi.org/10.1080/17441690903334232

Chapman, D. J., Morel, K., Anderson, A. K., Damio, G., & Pérez-Escamilla, R. (2010). Breastfeeding Peer Counseling: From Efficacy Through Scale-Up. *J Ournal of Human Lactation*, *26*(3), 314–326. https://doi.org/10.1177/0890334410369481

Cherrington, A., Ayala, G. X., Amick, H., Scarinci, I., Allison, J., & Corbie-Smith, G. (2008). Applying the Community Health Worker Model to Diabetes Management: Using Mixed Methods to Assess Implementation and Effectiveness. *Journal of Health Care for the Poor and Underserved*, *19*(4), 1044–1059.

Christopher, J. B., Le May, A., Lewin, S., & Ross, D. A. (2011). Thirty years after Alma-Ata: a systematic review of the impact of community health workers delivering curative interventions against malaria, pneumonia and diarrhoea on child mortality and morbidity in sub-Saharan Africa. *Human Resources for Health*, *9*, 27. https://doi.org/10.1186/1478-4491-9-27

Corley, A. G., Thornton, C. P., & Glass, N. E. (2016). The Role of Nurses and Community Health Workers in Confronting Neglected Tropical Diseases in Sub-Saharan Africa: A Systematic Review. *PLoS Neglected Tropical Diseases*. https://doi.org/10.1371/journal.pntd.0004914

Corluka, A., Walker, D. G., Lewin, S., Glenton, C., & Scheel, I. B. (2009). Are vaccination programmes delivered by lay health workers cost-effective? A systematic review. *Human Resources for Health*, *7*, 81. https://doi.org/10.1186/1478-4491-7-81

Costa, E. F., Guerra, P. H., Santos, T. I. dos, & Florindo, A. A. (2015). Systematic review of physical activity promotion by community health workers. *Preventive Medicine*, *81*, 114–121. https://doi.org/10.1016/j.ypmed.2015.08.007

Dale, J., Caramlau, I., Lindenmeyer, A., & Williams, S. M. (2008). Peer support telephone call interventions for improving health. *Cochrane Database of Systematic Reviews*, (1), CD006903. https://doi.org/10.1002/14651858.CD006903.pub2.www.cochranelibrary.com

Darmstadt, G. L., Lee, A. C. C., Cousens, S., Sibley, L. M., Bhutta, Z. A., Donnay, F., … Lawn, J. E. (2009). 60 Million non-facility births: Who can deliver in community settings to reduce intrapartum-related deaths? *International Journal of Gynecology and Obstetrics*, *107*(SUPPL.). https://doi.org/10.1016/j.ijgo.2009.07.010

Dawson, A. J., Brodie, P., Copeland, F., Rumsey, M., & Homer, C. S. E. (2014). Collaborative approaches towards building midwifery capacity in low income countries: A review of experiences. *Midwifery*, *30*(4), 391–402. https://doi.org/10.1016/j.midw.2013.05.009

Dawson, A. J., Buchan, J., Duffield, C., Homer, C. S. E., & Wijewardena, K. (2014). Task shifting and sharing in maternal and reproductive health in low-income countries: A narrative synthesis of current evidence. *Health Policy and Planning*, *29*(3), 396–408. https://doi.org/10.1093/heapol/czt026

de Oliveira Castro, T. T., & Zucki, F. (2015). Capacitação do Agente Comunitário de Saúde na saúde auditiva infantil: perspectivas atuais. *CoDAS*, *27*(6), 616–622. https://doi.org/10.1590/2317-1782/20152014223

Ehiri, J. E., Gunn, J. K. L., Center, K. E., Li, Y., Rouhani, M., & Ezeanolue, E. E. (2014). Training and deployment of lay refugee/internally displaced persons to provide basic health services in camps: a systematic review. *Global Health Action*, *7*, 23902. https://doi.org/10.3402/gha.v7.23902

Flynn, D. E., Johnson, C., Sands, A., Wong, V., Figueroa, C., & Baggaley, R. (2017). Can trained lay providers perform HIV testing services? A review of national HIV testing policies. *BMC Research Notes*, *10*(1), 20. https://doi.org/10.1186/s13104-016-2339-1

Fulton, B. D., Scheffler, R. M., Sparkes, S. P., Auh, E. Y., Vujicic, M., & Soucat, A. (2011). Health workforce skill mix and task shifting in low income countries: a review of recent evidence. *Human Resources for Health*, *9*(1), 1. https://doi.org/10.1186/1478-4491-9-1

Gibbons, M. C., & Tyrus, N. C. (2007). Systematic Review of U.S.-Based Randomized Controlled Trials Using Community Health Workers. *Progress in Community Health Partnerships: Research, Education and Action*, *1*(4), 371–381.

Gilmore, B., & McAuliffe, E. (2013). Effectiveness of community health workers delivering preventive interventions for maternal and child health in low- and middle-income countries: a systematic review. *BMC Public Health*, *13*(1), 847. https://doi.org/10.1186/1471-2458-13-847

Giugliani, C., Harzheim, E., Duncan, M. S., & Duncan, B. B. (2011). Effectiveness of community health workers in Brazil: a systematic review. *The Journal of Ambulatory Care Management*, *34*(4), 326–338. https://doi.org/10.1097/JAC.0b013e31822cbdfd [doi]

Glenton, C., Khanna, R., Morgan, C., & Nilsen, E. S. (2013). The effects, safety and acceptability of compact, pre-filled, autodisable injection devices when delivered by lay health workers. *Tropical Medicine and International Health*, *18*(8), 1002–1016. https://doi.org/10.1111/tmi.12126

Glenton, C., Scheel, I. B., Lewin, S., & Swingler, G. H. (2011). Can lay health workers increase the uptake of childhood immunisation? Systematic review and typology. *Tropical Medicine and International Health*, *16*(9), 1044–1053. https://doi.org/10.1111/j.1365-3156.2011.02813.x

Gogia, S., & Sachdev, H. P. S. (2016). Home-based neonatal care by community health workers for preventing mortality in neonates in low- and middle-income countries: a systematic review. *Journal of Perinatology*, *36*(S1), S55–S73. https://doi.org/10.1038/jp.2016.33

Gogia, S., Ramji, S., Gupta, P., Gera, T., Shah, D., Mathew, J. L., … Panda, R. (2011). Community Based Newborn Care: A Systematic Review and Meta-analysis of Evidence. *Indian Pediatrics*, *48*(7), 537–46.

Gogia, S., & Sachdev, H. S. (2010). Home visits by community health workers to prevent neonatal deaths in developing countries: a systematic review. *Bulletin of the World Health Organization*, *88*(9), 658–666. https://doi.org/10.2471/BLT.09.069369

Hall, B. J., Rachel, K. S., Mellanye, B., Sze, L., Qingyan, T., Doherty, M., … Hall, B. J. (2017). Barriers and Facilitators to Interventions Improving Retention in HIV Care : A Qualitative Evidence Meta-Synthesis. *AIDS and Behavior*, *21*(6), 1755–1767. https://doi.org/10.1007/s10461-016-1537-0

Henriques Camelo, S. H., Galon, T., & Palucci Marziale, M. H. (2012). Work Related Illness and Health Management Strategies Among Community Health Workers. *Rev Enferm UERJ*, *20*, 661–667.

Hill, Z., Dumbaugh, M., Benton, L., Ka, K., Strachan, D. L., Asbroek, A., … Meek, S. (2014). Supervising communtiy health workers n low-income countries- a review of impact and implementation issues. *Global Health Action*, *7*, 1–10.

Hoeft, T. J., Fortney, J. C., Patel, V., & Unützer, J. (2016). Task-Sharing Approaches to Improve Mental Health Care in Rural and Other Low-Resource Settings: A Systematic Review. *The Journal of Rural Health*, *0*, 1–15. https://doi.org/10.1111/jrh.12229

Horey, D., Street, A. F., O’Connor, M., Peters, L., & Lee, S. F. (2015). Training and supportive programs for palliative care volunteers in community settings. *The Cochrane Database of Systematic Reviews*, *7*(7), CD009500. https://doi.org/10.1002/14651858.CD009500.pub2

Hou, S.-I., & Roberson, K. (2015). A systematic review on US-based community health navigator (CHN) interventions for cancer screening promotion--comparing community- versus clinic-based navigator models. *Journal of Cancer Education : The Official Journal of the American Association for Cancer Education*, *30*(1), 173–186. https://doi.org/10.1007/s13187-014-0723-x

Hunt, C. W., Grant, J. S., & Appel, S. J. (2011). An integrative review of community health advisors in type 2 diabetes. *Journal of Community Health*, *36*(5), 883–893. https://doi.org/10.1007/s10900-011-9381-7

Islam, N. S., Zanowiak, J. M., Riley, L., Nadkarni, S. K., Kwon, S. C., & Trinh-Shevrin, C. (2015). Characteristics of Asian American, Native Hawaiian, and Pacific Islander Community Health Worker Programs: A Systematic Review. *Journal of Health Care for the Poor and Underserved*, *26*(2), 238–268.

Jaskiewicz, W., & Tulenko, K. (2012). Increasing community health worker productivity and effectiveness: a review of the influence of the work environment. *Human Resources for Health*, *10*(1), 38. https://doi.org/10.1186/1478-4491-10-38

Jones, C. C. G., Jomeen, J., & Hayter, M. (2014). The impact of peer support in the context of perinatal mental illness: A meta-ethnography. *Midwifery*, *30*(5), 491–498. https://doi.org/http://dx.doi.org/10.1016/j.midw.2013.08.003

Kabaghe, A. N., Visser, B. J., Spijker, R., Phiri, K. S., Grobusch, M. P., & van Vugt, M. (2016). Health workers’ compliance to rapid diagnostic tests (RDTs) to guide malaria treatment: a systematic review and meta-analysis. *Malaria Journal*, *15*(1), 163. https://doi.org/10.1186/s12936-016-1218-5

Källander, K., Tibenderana, J. K., Akpogheneta, O. J., Strachan, D. L., Hill, Z., Asbroek, A., … Meek, S. R. (2013). Mobile health (mhealth) approaches and lessons for increased performance and retention of community health workers in lowand middle-income countries: A review. *Journal of Medical Internet Research*, *15*(1). https://doi.org/10.2196/jmir.2130

Kamal-Yanni, M. M., Potet, J., & Saunders, P. M. (2012). Scaling-up malaria treatment: a review of the performance of different providers. *Malaria Journal*, *11*(1), 414. https://doi.org/10.1186/1475-2875-11-414

Kane, S. S., Gerretsen, B., Scherpbier, R., Dal Poz, M., & Dieleman, M. (2010). A realist synthesis of randomised control trials involving use of community health workers for delivering child health interventions in low and middle income countries. *BMC Health Services Research*, *10*(1), 286. https://doi.org/10.1186/1472-6963-10-286

Kane, S., Kok, M., Ormel, H., Otiso, L., Sidat, M., Namakhoma, I., … de Koning, K. (2016). Limits and opportunities to community health worker empowerment: A multi-country comparative study. *Social Science and Medicine*, *164*, 27–34. https://doi.org/10.1016/j.socscimed.2016.07.019

Kaunonen, M., Hannula, L., & Tarkka, M. T. (2012). A systematic review of peer support interventions for breastfeeding. *Journal of Clinical Nursing*, *21*(13–14), 1943–1954. https://doi.org/10.1111/j.1365-2702.2012.04071.x

Kew, K. M., Carr, R., & Crossingham, I. (2016). Lay-led and peer support interventions for adolescents with asthma (Review). *Cochrane Database of Systematic Reviews*, (8). https://doi.org/10.1002/14651858.CD012331

Kok, M. C., Dieleman, M., Taegtmeyer, M., Broerse, J. E. W., Kane, S. S., Ormel, H., … De Koning, K. A. M. (2015). Which intervention design factors influence performance of community health workers in low- and middle-income countries? A systematic review. *Health Policy and Planning*, *30*(9), 1207–1227. https://doi.org/10.1093/heapol/czu126

Kok, M. C., Kane, S. S., Tulloch, O., Ormel, H., Theobald, S., Dieleman, M., … De Koning, K. A. M. (2015). How does context influence performance of community health workers in low- and middle-income countries? Evidence from the literature. *Health Res Policy Syst*, *13*(1), 13. https://doi.org/10.1186/s12961-015-0001-3

Koon, A. D., Goudge, J., & Norris, S. A. (2013). A review of generalist and specialist community health workers for delivering adolescent health services in sub-Saharan Africa. *Human Resources for Health [Electronic Resource]*, *11*, 54. https://doi.org/http://dx.doi.org/10.1186/1478-4491-11-54

Kredo, T., Adeniyi, F. B., Bateganya, M., & Pienaar, E. D. (2014). Task shifting from doctors to non-doctors for initiation and maintenance of antiretroviral therapy ( Review ). *Cochrane Database of Systematic Reviews*, (7). https://doi.org/10.1002/14651858.CD007331.pub3.www.cochranelibrary.com

Lassi, Z. S., & Bhutta, Z. A. (2015). Community-based intervention packages for reducing maternal and neonatal morbidity and mortality and improving neonatal outcomes (Review). *Cochrane Database of Systematic Reviews*, (3), CD007754. https://doi.org/10.1002/14651858.CD007754.pub3.www.cochranelibrary.com

Lee, A. C. C., Chandran, A., Herbert, H. K., Kozuki, N., Markell, P., Shah, R., … Baqui, A. H. (2014). Treatment of Infections in Young Infants in Low- and Middle-Income Countries: A Systematic Review and Meta-analysis of Frontline Health Worker Diagnosis and Antibiotic Access. *PLoS Medicine*, *11*(10). https://doi.org/10.1371/journal.pmed.1001741

Lewin, S., Munabi-Babigumira, S., Glenton, C., Daniels, K., Bosch-Capblanch, X., van Wyk, B. E., … Scheel, I. B. (2010). Lay health workers in primary and community health care for maternal and child health and the management of infectious diseases. *Cochrane Database Syst Rev*, *3*(3), CD004015. https://doi.org/10.1002/14651858.CD004015.pub3

Little, T. V., Wang, M. L., Castro, E. M., Jimenez, J., & Rosal, M. C. (2014). Community Health Worker Interventions for Latinos With Type 2 Diabetes: a Systematic Review of Randomized Controlled Trials. *Current Diabetes Reports*, *14*(12), 1–16. https://doi.org/10.1007/s11892-014-0558-1

Lizarondo, L., Kumar, S., Hyde, L., & Skidmore, D. (2010). Allied health assistants and what they do: A systematic review of the literature. *Journal of Multidisciplinary Healthcare*, *3*, 143–153. https://doi.org/10.2147/JMDH.

Loures, L. F., & Silva, M. C. de S. (2010). Interface between the work of the community health agent and physiotherapist in the basic health care. *Ciência & Saúde Coletiva*, *15*(4), 2155–2164. https://doi.org/10.1590/S1413-81232010000400029

Ma, Q., Tso, L. S., Rich, Z. C., Hall, B. J., Beanland, R., Li, H., … Tucker, J. D. (2016). Barriers and facilitators of interventions for improving antiretroviral therapy adherence: a systematic review of global qualitative evidence. *Journal of the International AIDS Society*, *19*(1), 1–13. https://doi.org/10.7448/IAS.19.1.21166

Malarcher, S., Meirik, O., Lebetkin, E., Shah, I., Spieler, J., & Stanback, J. (2011). Provision of DMPA by community health workers: What the evidence shows. *Contraception*, *83*(6), 495–503. https://doi.org/10.1016/j.contraception.2010.08.013

Maravilla, J. C., Betts, K. S., Abajobir, A. A., Couto e Cruz, C., & Alati, R. (2016). The Role of Community Health Workers in Preventing Adolescent Repeat Pregnancies and Births. *Journal of Adolescent Health*. Elsevier Inc. https://doi.org/10.1016/j.jadohealth.2016.05.011

Martínez-Donate, A. P. (2009). Using lay health advisors to promote breast and cervical cancer screening among Latinas: a review. *WMJ : Official Publication of the State Medical Society of Wisconsin*, *108*(5), 259–62. Retrieved from http://www.ncbi.nlm.nih.gov/pubmed/19743759

McCollum, R., Gomez, W., Theobald, S., & Taegtmeyer, M. (2016). How equitable are community health worker programmes and which programme features influence equity of community health worker services? A systematic review. *BMC Public Health*, *16*, 419. https://doi.org/10.1186/s12889-016-3043-8

Mdege, N. D., Chindove, S., & Ali, S. (2013). The effectiveness and cost implications of task-shifting in the delivery of antiretroviral therapy to HIV-infected patients: A systematic review. *Health Policy and Planning*, *28*(3), 223–236. https://doi.org/10.1093/heapol/czs058

Mercer, C., Byrth, J., & Jordan, Z. (2014). The experiences of Aboriginal health workers and non‐Aboriginal health professionals working collaboratively in the delivery of health care to Aboriginal Australians: a systematic review. *JBI Database of Systematic Reviews and Implementation Reports*, *12*(3), 274–418. https://doi.org/10.11124/jbisrir-2014-1105

Miyake, S., Speakman, E. M., Currie, S., & Howard, N. (2017). Community midwifery initiatives in fragile and conflict-affected countries: A scoping review of approaches from recruitment to retention. *Health Policy and Planning*. https://doi.org/10.1093/heapol/czw093

Mutamba, B. B., van Ginneken, N., Smith Paintain, L., Wandiembe, S., & Schellenberg, D. (2013). Roles and effectiveness of lay community health workers in the prevention of mental, neurological and substance use disorders in low and middle income countries: a systematic review. *BMC Health Serv Res*, *13*, 412. https://doi.org/10.1186/1472-6963-13-412

Mwai, G., Mburu, G., Torpey, K., Frost, P., Ford, N., & Seeley, J. (2013). Role and outcomes of community health workers in HIV care in sub-Saharan Africa: a systematic review. *Journal of the International AIDS Society*, *16*, 1–14. https://doi.org/10.7448/ias.16.1.18586

Noordam, A. C., Barberá Laínez, Y., Sadruddin, S., van Heck, P. M., Chono, A. O., Acaye, G. L., … Källander, K. (2015). The use of counting beads to improve the classification of fast breathing in low-resource settings: a multi-country review. *Health Policy and Planning*, *30*(6), 696–704. https://doi.org/10.1093/heapol/czu047

Norris, S. L., Chowdhury, F. M., Le, K. Van, Horsley, T., Brownstein, J. N., Zhang, X., … Satterfield, D. (2006). Effectiveness of community health workers in the care of persons with diabetes Effectiveness of community health workers in the care of persons with diabetes. *Diabetic Medicine*, *23*, 544–556. https://doi.org/10.1111/j.1464-5491.2006.01845.x

Oyo-Ita, A., Wiysonge, C. S., Oringanje, C., Nwachukwu, C. E., Oduwole, O., & Meremikwu, M. M. (2016). Interventions for improving coverage of childhood immunisation in low- and middle-income countries. *Cochrane Database of Systematic Reviews*, (7). https://doi.org/10.1002/14651858.CD008145.pub3.www.cochranelibrary.com

Paintain, L. S., Willey, B., Kedenge, S., Sharkey, A., Kim, J., Buj, V., … Ngongo, N. (2014). Community health workers and stand-alone or integrated case management of malaria: A systematic literature review. *American Journal of Tropical Medicine and Hygiene*, *91*(3), 461–470. https://doi.org/10.4269/ajtmh.14-0094

Pallas, S. W., Minhas, D., Pérez-Escamilla, R., Taylor, L., Curry, L., Bradley, E. H., … Bradley, E. H. (2013). Community health workers in low- and middle-income countries: What do we know about scaling up and sustainability? *American Journal of Public Health*, *103*(7), 74–82. https://doi.org/10.2105/AJPH.2012.301102

Palmas, W., March, D., Darakjy, S., Findley, S. E., Teresi, J., Carrasquillo, O., & Luchsinger, J. A. (2015). Community Health Worker Interventions to Improve Glycemic Control in People with Diabetes: A Systematic Review and Meta-Analysis. *Journal of General Internal Medicine*, 1004–1012. https://doi.org/10.1007/s11606-015-3247-0

Patel, A. R., & Nowalk, M. P. (2010). Expanding immunization coverage in rural India: A review of evidence for the role of community health workers. *Vaccine*, *28*(3), 604–613. https://doi.org/10.1016/j.vaccine.2009.10.108

Pereira, I. C., & Oliveira, M. A. C. (2013). O trabalho do agente comunitário na promoção da saúde : revisão integrativa da literatura. *Revista Brasileira de Enfermagem*, *66*(3), 412–419. https://doi.org/10.1590/S0034-71672013000300017

Petersen, I., Fairall, L., Egbe, C. O., & Bhana, A. (2014). Optimizing lay counsellor services for chronic care in South Africa: A qualitative systematic review. *Patient Education and Counseling*, *95*(2), 201–210. https://doi.org/10.1016/j.pec.2014.02.001

Postma, J., Karr, C., & Kieckhefer, G. (2009). Community health workers and environmental interventions for children with asthma: a systematic review. *The Journal of Asthma : Official Journal of the Association for the Care of Asthma*, *46*(1), 564–576. https://doi.org/10.1080/02770900902912638

Prost, A., Colbourn, T., Seward, N., Azad, K., Coomarasamy, A., Copas, A., … Costello, A. (2013). Women’s groups practising participatory learning and action to improve maternal and newborn health in low-resource settings: a systematic review and meta-analysis. *The Lancet*, *381*(9879), 1736–1746. https://doi.org/10.1016/S0140-6736(13)60685-6

Rahman, A., Fisher, J., Bower, P., Luchters, S., Tran, T., & Yasamy, M. T. (2013). Interventions for common perinatal mental disorders in women in low- and middle-income countries : a systematic review and meta-analysis. *Bulletin of the World Health Organization*, *91*, 593–601.

Raphael, J. L., Rueda, A., Lion, K. C., & Giordano, T. P. (2013). The role of lay health workers in pediatric chronic disease: A systematic review. *Academic Pediatrics*, *13*(5), 408–420. https://doi.org/10.1016/j.acap.2013.04.015

Reisman, J., Arlington, L., Jensen, L., Louis, H., Suarez-Rebling, D., & Nelson, B. D. (2016). Newborn Resuscitation Training in Resource-Limited Settings: A Systematic Literature Review. *Pediatrics*, *138*(2), e20154490-. https://doi.org/10.1542/peds.2015-4490

Rhodes, S. D., Foley, K. L., Zoneta, C. S., & Bloom, F. R. (2007). Lay Health Advisor Interventions Among Hispanics/Latinos: A Qualitative Systematic Review. *American Journal of Preventive Medicine*, *33*(5), 418–427.

Ribeiro Sarmento, D. (2014). Traditional Birth Attendance (TBA) in a health system: what are the roles, benefits and challenges: A case study of incorporated TBA in Timor-Leste. *Asia Pacific Family Medicine*, *13*(1), 12. https://doi.org/10.1186/s12930-014-0012-1

Ruizendaal, E., Dierickx, S., Peeters Grietens, K., Schallig, H. D. F. H., Pagnoni, F., & Mens, P. F. (2014). Success or failure of critical steps in community case management of malaria with rapid diagnostic tests: a systematic review. *Malaria Journal*, *13*, 229. https://doi.org/10.1186/1475-2875-13-229

Sazawal, S., & Black, R. E. (2003). Effect of pneumonia case management on mortality in neonates, infants, and preschool children: A meta-analysis of community-based trials. *Lancet Infectious Diseases*, *3*(9), 547–556. https://doi.org/10.1016/S1473-3099(03)00737-0

Schneider, H., Okello, D., & Lehmann, U. (2016). The global pendulum swing towards community health workers in low- and middle-income countries: a scoping review of trends, geographical distribution and programmatic orientations, 2005 to 2014. *Human Resources for Health*, *14*(1), 65. https://doi.org/10.1186/s12960-016-0163-2

Scott, V. K., Gottschalk, L. B., Wright, K. Q., Twose, C., Bohren, M. A., Schmitt, M. E., & Ortayli, N. (2015). Community Health Workers’ Provision of Family Planning Services in Low- and Middle-Income Countries: A Systematic Review of Effectiveness. *Studies in Family Planning*, *46*(3), 241–261. https://doi.org/10.1111/j.1728-4465.2015.00028.x

Shommu, N. S., Ahmed, S., Rumana, N., Barron, G. R. S., McBrien, K. A., & Turin, T. C. (2016). What is the scope of improving immigrant and ethnic minority healthcare using community navigators: A systematic scoping review. *International Journal for Equity in Health*, *15*(1), 6. https://doi.org/10.1186/s12939-016-0298-8

Sibley, L. M., & Sipe, T. A. (2006). Transition to skilled birth attendance: Is there a future role for trained traditional birth attendants? *Journal of Health, Population and Nutrition*, *24*(4), 472–478.

Sibley, L. M., Sipe, T. A., & Barry, D. (2012). Traditional birth attendant training for improving health behaviours and pregnancy outcomes. *Cochrane Database of Systematic Reviews*, *8*(8). https://doi.org/10.1002/14651858.CD005460.pub3.Copyright

Silveira Feyer, I. S., Monticelli, M., Volkmer, C., & Burigo, R. A. (2013). Brazilian Scientific Publications of Obstetrical Nurses on Home Delivery : Systematic Literature Review 1 Publicações Científicas Brasileiras De Enfermeiras Obstétricas Sobre Parto Domiciliar : Revisão Sistemática Publicaciones Científicas Brasileiras De. *Text Context Nursing*, *22*(1), 247–256.

Singla, D. R., Kohrt, B. A., Murray, L. K., Anand, A., Chorpita, B. F., & Patel, V. (2017). Psychological Treatments for the World : Lessons from Low- and Middle-Income Countries. *Annual Review of Clinical Psychology*, (13), 149–81.

Small, N., Blickem, C., Blakeman, T., Panagioti, M., Chew-Graham, C. a, & Bower, P. (2013). Telephone based self-management support by “lay health workers” and “peer support workers” to prevent and manage vascular diseases: a systematic review and meta-analysis. *BMC Health Services Research*, *13*, 533. https://doi.org/10.1186/1472-6963-13-533

Smith, H. J., Colvin, C. J., Richards, E., Roberson, J., Sharma, G., Thapa, K., & G??lmezoglu, A. M. (2016). Programmes for advance distribution of misoprostol to prevent post-partum haemorrhage: A rapid literature review of factors affecting implementation. *Health Policy and Planning*. https://doi.org/10.1093/heapol/czv012

South, J., Meah, A., Bagnall, A.-M., & Jones, R. (2013). Dimensions of lay health worker programmes: results of a scoping study and production of a descriptive framework. *Global Health Promotion*, *20*(1), 5–15. https://doi.org/10.1177/1757975912464248

Stacciarini, J.-M. R., Rosa, A., Ortiz, M., Munari, D. B., Uicab, G., & Balam, M. (2012). Promotoras in Mental Health. *Family & Community Health*, *35*(2), 92–102. https://doi.org/10.1097/FCH.0b013e3182464f65

Stanhope, J., & Pearce, C. (2013). Role, implementation, and effectiveness of advanced allied health assistants: A systematic review. *Journal of Multidisciplinary Healthcare*, *6*, 423–434. https://doi.org/10.2147/JMDH.S50185

Sunguya, B. F., Mlunde, L. B., Ayer, R., & Jimba, M. (2017). Towards eliminating malaria in high endemic countries: the roles of community health workers and related cadres and their challenges in integrated community case management for malaria: a systematic review. *Malaria Journal*, *16*(1), 10. https://doi.org/10.1186/s12936-016-1667-x

Tso, L. S., Best, J., Beanland, R., Doherty, M., Lackey, M., Ma, Q., … Tucker, J. D. (2016). Facilitators and Barriers in HIV Linkage to Care Interventions: Qualitative Evidence Review. *AIDS*, *30*(10), 1639–1653. https://doi.org/10.1097/QAD.0000000000001101.Facilitators

van Ginneken, N., Tharyan, P., Lewin, S., Rao, G., Meera, S., Pian, J., … Patel, V. (2013). Non-Specialist Health Worker Interventions for the Care of Mental, Neurological, and Substance-Abuse Disorders in Low- and Middle-Income Countries. *Cochrane Database of Systematic Reviews*, *11*(CD009149). https://doi.org/10.3109/01612840.2015.1128299

Vaughan, K., Kok, M. C., Witter, S., & Dieleman, M. (2015). Costs and cost-effectiveness of community health workers: evidence from a literature review. *Human Resources for Health*, *13*(1), 71. https://doi.org/10.1186/s12960-015-0070-y

Viswanathan, M., Kraschnewski, J., Nishikawa, B., Morgan, L. C., Thieda, P., Honeycutt, A., … Jonas, D. (2009). *Outcomes of community health worker interventions.* *Evidence Report/Aechnology assessment*. Retrieved from http://www.ncbi.nlm.nih.gov/books/NBK44601/

Vouking, M. Z., Tamo, V. C., & Mbuagbaw, L. (2013). The impact of community health workers (CHWs) on Buruli ulcer in sub-Saharan Africa: a systematic review. *The Pan African Medical Journal*, *15*, 19. https://doi.org/10.11604/pamj.2013.15.19.1991

Wadler, B. M., Judge, C. M., Prout, M., Allen, J. D., & Geller, A. C. (2011). Improving breast cancer control via the use of community health workers in South Africa: A critical review. *Journal of Oncology*, *2011*(Table 1). https://doi.org/10.1155/2011/150423

Wahlbeck, K., Cresswell, J., Peija, S., & Parkkonen, J. (2017). Interventions to mitigate the effects of poverty and inequality on mental health. *Social Psychiatry and Psychiatric Epidemiology*, *52*(5), 505–514. https://doi.org/10.1007/s00127-017-1370-4

Wells, K. J., Luque, J. S., Miladinovic, B., Vargas, N., Asvat, Y., Roetzheim, R. G., & Kumar, A. (2011). Do community health worker interventions improve rates of screening mammography in the United States? A systematic review. *Cancer Epidemiology, Biomarkers & Prevention*, *20*(8), 1580–98. https://doi.org/10.1158/1055-9965.EPI-11-0276

WHO, & Global Health Workforce Alliance. (2010). *Global Experience of Community Health Workers for Delivery of Health Related Millennium Development Goals*. Retrieved from http://www.who.int/workforcealliance/knowledge/publications/alliance/Global_CHW_web.pdf

Wilson, a., Gallos, I. D., Plana, N., Lissauer, D., Khan, K. S., Zamora, J., … Coomarasamy, A. (2011). Effectiveness of strategies incorporating training and support of traditional birth attendants on perinatal and maternal mortality: meta-analysis. *BMJ*, *343*(dec01 1), d7102–d7102. https://doi.org/10.1136/bmj.d7102

Winch, P. J., Gilroy, K. E., Wolfheim, C., Starbuck, E. S., Young, M. W., Walker, L. D., & Black, R. E. (2005). Intervention models for the management of children with signs of pneumonia or malaria by community health workers. *Health Policy and Planning*, *20*(4), 199–212. https://doi.org/10.1093/heapol/czi027

Wouters, E., Van Damme, W., van Rensburg, D., Masquillier, C., & Meulemans, H. (2012). Impact of community-based support services on antiretroviral treatment programme delivery and outcomes in resource-limited countries: a synthetic review. *BMC Health Services Research*, *12*(1), 194. https://doi.org/10.1186/1472-6963-12-194

Wu, S., Roychowdhury, I., & Khan, M. (2017). Evaluating the impact of healthcare provider training to improve tuberculosis management: a systematic review of methods and outcome indicators used. *International Journal of Infectious Diseases*. International Society for Infectious Diseases. https://doi.org/10.1016/j.ijid.2016.11.421

Zhou, K., Fitzpatrick, T., Walsh, N., Kim, J. Y., Chou, R., Lackey, M., … Tucker, J. D. (2016). Articles Interventions to optimise the care continuum for chronic viral hepatitis: a systematic review and meta-analyses. *The Lancet Infectious Diseases*, *16*(12), 1409–1422. https://doi.org/10.1016/S1473-3099(16)30208-0

Zulu, J. M., Kinsman, J., Michelo, C., & Hurtig, A. K. (2014). Integrating national community-based health worker programmes into health systems: a systematic review identifying lessons learned from low- and middle-income countries. *BMC Public Health*, *14*(987), 1–17. Retrieved from http://onlinelibrary.wiley.com/o/cochrane/cldare/articles/DARE-12014059364/frame.html

**Excluded articles (n=32)**

These article titles and abstracts were retained from title and abstract review stage to full text review stage, at which point they were excluded. The reasons for exclusion were:

- No full text available (Austin 2012; Borda-Olivas et al. 2012; Ehiri et al. 2016; Macuacua et al. 2015; Musa et al. 2014; Potter 2011)
- Not a review (Kaur 2016; Kiarie Festin 2015; Lassi et al. 2016; Spencer, Gunter & Palmisano 2010; Trivedi 2016; Wagoner, Downs & Mario 2015)
- Not focused on community based practitioners (Awor et al. 2015; Bailey, et al. 2016; Banda, 2016; Colvin et al. 2013; Cowley et al. 2015; Glenton, Lewin & Scheel 2011; Kluthcovsky & Takayanagui 2006; Leech et al. 2007; Leeman et al. 2015; Legg Lynn et al. 2011; Machado et al. 2016; Munn, Tufanaru & Aromataris 2013; Ngidi et al. 2017; Renfrew et al. 2014; Renner, Brahmi & Kapp 2013; Shaban & Leap 2012; van der Heijden, Abrahams & Sinclair 2017; Wahedi 2016; West, Homer & Dawson 2016; Zhang, et al. 2016)

Austin, A. (2012). The impact of training traditional Birth attendants: A narrative systematic review. *Qjm*, *105*(6), 601.

Awor, P., Miller, J., & Peterson, S. (2014). Systematic literature review of integrated community case management and the private sector in Africa: Relevant experiences and potential next steps. *Journal of Global Health*, *4*(2), 20414.

Bailey, C., Blake, C., Schriver, M., Cubaka, V. K., Thomas, T., & Martin Hilber, A. (2016). A systematic review of supportive supervision as a strategy to improve primary healthcare services in Sub-Saharan Africa. *International Journal of Gynecology and Obstetrics*, *132*(1), 117–125.

Banda, H., Robinson, R., Thomson, R., Squire, S. B., & Mortimer, K. (2016). The “Practical Approach to Lung Health” in sub-Saharan Africa: a systematic review. The International Journal of Tuberculosis and Lung Disease, 20(4), 552–9.

Borda-Olivas, A., Palma-Ruiz, M., Drasbec, C., & Sarria-Santamera, A. (2012). Community Health Workers: A literature review in some countries of South America. *Value in Health*, *15*, 27–28.

Bornstein, V. J., & Stotz, E. N. (2008). Concepções que integram a formação e o processo de trabalho dos agentes comunitários de saúde: uma revisão da literatura. *Ciência & Saúde Coletiva*, *13*(1), 259–268.

Colvin, C. J., de Heer, J., Winterton, L., Mellenkamp, M., Glenton, C., Noyes, J., … Rashidian, A. (2013). A systematic review of qualitative evidence on barriers and facilitators to the implementation of task-shifting in midwifery services. *Midwifery*, *29*(10), 1211–1221.

Cowley, S., Whittaker, K., Malone, M., Donetto, S., Grigulis, A., & Maben, J. (2015). Why health visiting? Examining the potential public health benefits from health visiting practice within a universal service: A narrative review of the literature. *International Journal of Nursing Studies*, *52*(1), 465–480.

Ehiri, I. O. Asaolu, H. M. Dreifuss, J. C. Ehiri and C. Valencia. (2016). Community health workers and prevention of chronic diseases in low-and middle-income countries: A systematic review. Annals of Global Health, 82, (3): 414-415.

Glenton, C., Lewin, S., & Scheel, I. B. (2011). Still too little qualitative research to shed light on results from reviews of effectiveness trials: a case study of a Cochrane review on the use of lay health workers. *Implementation Science : IS*, *6*(1), 53.

Kaur, M. (2016). Community Health Workers — Birth of a New Profession. *Generations: Journal of the American Society on Aging*, *40*(1), 56–64.

Kiarie, J., & Festin, M. (2015). WHO guidelines on task shifting/sharing to improve access to contraception. *International Journal of Gynecology and Obstetrics*, *131*(Suppl. 5), E1–E71.

Kluthcovsky, A. C. G. C., & Takayanagui, A. M. M. (2006). Community health agent: a literature review. *Rev Latino-Am Enfermagem*, *14*(6), 957–963.

Lassi, Z., Middleton, P., Bhutta, Z., & Crowther, C. (2016). Strategies for improving health care seeking for maternal and newborn illnesses in low and middle income countries: A systematic review and meta-analysis. *Journal of Paediatrics and Child Health*, 52, 3–28.

Leech, R., van Wyk, N. C., & Uys, C. J. E. (2007). The management of infant developmental needs by community nurses. Part 2: the development of guidelines for the support of community nurses in the management of infant developmental needs. *Curationis*, *30*(2), 104–12.

Leeman, J., Calancie, L., Hartman, M. A., Escoffery, C. T., Herrmann, A. K., Tague, L. E., … Samuel-Hodge, C. (2015). What strategies are used to build practitioners’ capacity to implement community-based interventions and are they effective?: a systematic review. *Implementation Science : IS*, *10*, 80.

Legg, L. a, Quinn, T. J., Mahmood, F., Weir, C. J., Tierney, J., Stott, D. J., … Langhorne, P. (2011). Non-pharmacological interventions for caregivers of stroke survivors ( Review ). *Cochrane* Database of Systematic Reviews, (10).

Machado, A. P., Lima, B. M., Laureano, M. G., Silva, P. H. B., Tardin, G. P., Reis, P. S., … D’Artibale, E. F. (2016). Educational strategies for the prevention of diabetes, hypertension, and obesity. Revista Da Associatio Medica Brasileira, 62(8), 800–808.

Macuacua, S., Sharma, S., Vala, A., Vidler, M., Nhama, A., Macete, E., … Von Dadelszen, P. (2015). Policy review on management of pre-eclampsia and eclampsia by community health workers in Mozambique. *International Journal of Gynecology and Obstetrics*, *131*, E338–E338.

Munn, Z., Tufanaru, C., & Aromataris, E. (2013). Recognition of the health assistant as a delegated clinical role and their inclusion in models of care: A systematic review and meta-synthesis of qualitative evidence. *International Journal of Evidence-Based Healthcare*, *11*(1), 3–19.

Musa, Z. Iliyasu, S. M. Yusuf and A. E. Uloko. (2014). Systematic review and metanalysis on community based interventions in tuberculosis care in developing countries. Niger J Med 23(2):103-17.

Ngidi, W. H., Naidoo, J. R., Ncama, B. P., Luvuno, Z. P. B., & Mashamba-Thompson, T. P. (2017). Mapping evidence of interventions and strategies to bridge the gap in the implementation of the prevention of mother-to-child transmission of HIV programme policy in sub-Saharan countries: A scoping review. African Journal of Primary Health Care and Family Medicine, 9(1).

Potter, C. L. (2011). Systematic literature review of the use of lay support models in postnatal depression. *European Psychiatry*, *26*, 1105.

Renfrew, M. J., McFadden, A., Bastos, M. H., Campbell, J., Channon, A. A., Cheung, N. F., … Declercq, E. (2014). Midwifery and quality care: Findings from a new evidence-informed framework for maternal and newborn care. *The Lancet 384* (9948): 1129-1145.

Renner, R. M., Brahmi, D., & Kapp, N. (2013). Who can provide effective and safe termination of pregnancy care? A systematic review. *BJOG: An International Journal of Obstetrics and Gynaecology*, *120*(1), 23–31.

Shaban, I., & Leap, N. (2012). A review of midwifery education curriculum documents in Jordan. *Women and Birth*, *25*(4), e47–e55.

Spencer, K. E. Gunter and G. Palmisano (2010). Community health workers and their value to social work. Soc Work 55(2):169-80.

Trivedi, D. (2016). Cochrane Review Summary: Community-based intervention packages for reducing maternal and neonatal morbidity and mortality and improving neonatal outcomes Primary Health Care Research & Development 17: 317–318.

van der Heijden, I., Abrahams, N., & Sinclair, D. (2017). Psychosocial group interventions to improve psychological well-being in adults living with HIV (Review). *Cochrane Database of Systematic Reviews*, (3).

Wagoner, K. G., Downs, M., Alonzo, J., Daniel-Ulloa, J., & Rhodes, S. D. (2015). Latino men’s qualitative perspectives on a lay health advisor intervention to promote their sexual health. *Health and Social Care in the Community*, *23*(3), 304–312.

West, F., Homer, C., & Dawson, A. (2015). Building midwifery educator capacity in teaching in low and lower-middle income countries. A review of the literature. *Midwifery*, *33*, 12–23.

Zhang, M., Yang, R., Wang, W., Gillespie, J., Clarke, S., & Yan, F. (2016). Job satisfaction of urban community health workers after the 2009 healthcare reform in China: A systematic review. *International Journal for Quality in Health Care*, *28*(1), 14–21.
